# Characterizing Neutrophil Subtypes in Cancer Using scRNA Sequencing Demonstrates the Importance of IL1β/CXCR2 Axis in Generation of Metastasis-specific Neutrophils

**DOI:** 10.1158/2767-9764.CRC-23-0319

**Published:** 2024-02-29

**Authors:** Rana Fetit, Alistair S. McLaren, Mark White, Megan L. Mills, John Falconer, Xabier Cortes-Lavaud, Kathryn Gilroy, Tamsin R.M. Lannagan, Rachel A. Ridgway, Colin Nixon, Varushka Naiker, Renee Njunge, Cassie J. Clarke, Declan Whyte, Kristina Kirschner, Rene Jackstadt, Jim Norman, Leo M. Carlin, Andrew D. Campbell, Owen J. Sansom, Colin W. Steele

**Affiliations:** 1 CRUK Scotland Institute, Glasgow, United Kingdom.; 2 School of Cancer Sciences, MVLS, University of Glasgow, Glasgow, United Kingdom.; 3 Beatson West of Scotland Cancer Centre, Glasgow, United Kingdom.; 4 Glasgow Royal Infirmary, Glasgow, United Kingdom.

## Abstract

Neutrophils are a highly heterogeneous cellular population. However, a thorough examination of the different transcriptional neutrophil states between health and malignancy has not been performed. We utilized single-cell RNA sequencing of human and murine datasets, both publicly available and independently generated, to identify neutrophil transcriptomic subtypes and developmental lineages in health and malignancy. Datasets of lung, breast, and colorectal cancer were integrated to establish and validate neutrophil gene signatures. Pseudotime analysis was used to identify genes driving neutrophil development from health to cancer. Finally, ligand–receptor interactions and signaling pathways between neutrophils and other immune cell populations in primary colorectal cancer and metastatic colorectal cancer were investigated. We define two main neutrophil subtypes in primary tumors: an activated subtype sharing the transcriptomic signatures of healthy neutrophils; and a tumor-specific subtype. This signature is conserved in murine and human cancer, across different tumor types. In colorectal cancer metastases, neutrophils are more heterogeneous, exhibiting additional transcriptomic subtypes. Pseudotime analysis implicates IL1β/CXCL8/CXCR2 axis in the progression of neutrophils from health to cancer and metastasis, with effects on T-cell effector function. We propose that the emergence of metastatic-specific neutrophil subtypes is driven by the IL1β/CXCL8/CXCR2 axis, with the evolution of different transcriptomic signals that impair T-cell function at the metastatic site. Thus, a better understanding of neutrophil transcriptomic programming could optimize immunotherapeutic interventions into early and late interventions, targeting different neutrophil states.

**Significance:**

We identify two recurring neutrophil populations and demonstrate their staged evolution from health to malignancy through the IL1β/CXCL8/CXCR2 axis, allowing for immunotherapeutic neutrophil-targeting approaches to counteract immunosuppressive subtypes that emerge in metastasis.

## Introduction

Neutrophils are short-lived cells released from the bone marrow in response to infection and inflammation and represent the most abundant circulating white blood cells. Traditionally thought of as terminally differentiated cells, neutrophils have been shown to demonstrate remarkable plasticity in response to different tissue environments (1), particularly to the tumor microenvironment (2). In previous studies, in murine models of human disease, we observed significant phenotypic differences between primary and metastatic tumors in response to inhibition of neutrophil populations using genetic and pharmacologic approaches. In both metastatic pancreatic and colorectal cancer models, we observed targeting neutrophil infiltration to metastases resulted in reduction of metastatic burden but with limited impact on primary tumor (PT) growth (3–5). These observations are in-keeping with other studies that have shown the potential of targeting metastasis-associated neutrophils therapeutically in murine models of cancer (6, 7). Oncogenic *KRAS* driver mutations in lung and colorectal cancers (8, 9) are thought to play a key role in driving neutrophil phenotype within tumors, with clear upregulation of neutrophil chemotactic protein production from metastatic lesions (10). Understanding the regulation of neutrophils within metastases will permit future therapeutic efforts, with the aim of promoting an antitumoral neutrophil phenotype. Indeed, recent work has shown the importance of antitumor neutrophils in clearing tumor cells during successful immunotherapy treatment (11, 12). Systemic neutrophilia and inflammation have repeatably been associated with poor outcomes in colorectal cancer as well as in other cancers (13). While these observations suggest the key role of neutrophils for promoting metastasis, dense neutrophil infiltration in the PT microenvironment also correlates with poor prognosis in colorectal cancer suggesting neutrophils have roles in cancer progression at both primary and metastatic sites (13). Overall, clear clinical and preclinical evidence exists for protumorigenic neutrophil populations, though awareness of neutrophils states is only just becoming recognized (14).

Indeed, several studies have utilized single-cell RNA sequencing (scRNA-seq) to delineate the immune cell populations infiltrating the tumor microenvironment in different cancers and their respective murine models, with a focus on macrophages and T-cell populations (15–20). However, to date, no study has thoroughly investigated neutrophil populations to specifically identify recurrent transcriptional subtypes in health and cancer. This underrepresentation of neutrophil populations in scRNA-seq datasets is largely owing to their short half-life, which dictates the fast processing of freshly procured samples, and the challenges in isolating adequate quality and quantity of RNA for downstream analysis, making neutrophils harder to capture using the common single-cell platforms. Moreover, Ficoll separation discards hypersegmented cells, making it difficult to isolate neutrophils from the blood (21). Furthermore, neutrophils and monocytes coexpress several antigens and produce similar effector molecules, proteins, chemokines, and cytokines, rendering them harder to identify or more likely to be mislabeled (22). However, combining datasets can enable analysis of this underrepresented cell type that has not been extensively evaluated before. Likewise, neutrophil phenotypic differences between normal and tumor-associated neutrophils have never been described at the single-cell level.

Therefore, we sought to assess the differences in neutrophil transcriptional phenotypes between healthy tissue, PT tissue, and liver metastatic (LM) tissue across different cancer types: lung, breast, and colorectal cancer. Neutrophils have been shown in these cancer types to influence outcomes, in both mice and humans (23). We hypothesized that neutrophils show plasticity and adaptation to their surroundings to support antitumorigenic or protumorigenic processes, with the metastatic site co-opting neutrophils to promote protumorigenic neutrophil function. We demonstrate using publicly available scRNA-seq datasets and data generated from colorectal cancer murine models that two main subsets of neutrophils can be identified in health and cancer. We identify the developmental trajectory of these cells and observed a heterogeneous group within LM tissue consistent with tissue-specific adaptation at the metastatic site. This study lends novel insights to neutrophil single-cell transcriptomic phenotypes and infers how these cells may be manipulated for therapeutic benefit in the future.

## Materials and Methods

### Processing Publicly Available Datasets

Datasets which had successfully captured and identified neutrophil clusters in their original publications were retrieved from the Gene Expression Omnibus (GEO) database and National Omics Encyclopaedia (Table 1) and processed using Seurat (version 4.3.0) on R (versions 3.17 and 4.1.1). Datasets were integrated by reciprocal principal component analysis (RPCA) using the IntegrateData function then scaled and normalized. Dimension reduction was performed using principal component analysis (PCA) followed by clustering using the FindNeighbors and FindClusters functions. Marker genes for individual clusters were determined using the FindAllMarkers function. Neutrophils were isolated by the authors using the cluster identities assigned in the meta-data and markers used in the original publications (Table 1). Datasets were integrated to establish and test neutrophil gene signatures using the AddModuleScore function. Pseudotime analysis was performed using Slingshot (version 2.8.0) to identify neutrophil lineages. Gene expression along the different trajectories was performed using TradeSeq (version 1.14.0). Gene set enrichment (GSE), Gene Ontology (GO), and Kyoto Encyclopedia of Genes and Genomes (KEGG) analyses were performed using ClusterProfiler (version 4.8.1) and EnrichR (version 3.2). Ligand-receptor (L-R) interactions and signaling pathways between neutrophils and other immune cell populations in primary and metastatic sites were investigated using CellChat (version 1.6.1). Software processing pipelines are listed in Table 2 and all relevant code can be accessed on Github (https://github.com/ranafetit/NeutrophilCharacterisation).

**TABLE 1:** Description of public datasets used in this study

Author	Accession number	Species	Tissue	Tumor	Description	Neutrophil identification method	Neutrophil Markers^a^
Zilionis et al., 2019	GSE127465	Human, Mouse	Blood, Lung	PT	Non–small cell lung cancer	Bayesian cell type classifier of CD45^+^ immune cell types and comparison cell states to published datasets	CXCL8, G0S2, CXCR2, S100A8, S100A9 FCGR3B, CSF3R, FFAR2
Grieshaber-Bouyer et al., 2021	GSE165276	Mouse	Bone marrow, Blood, Spleen	None	Healthy tissue	Sorting Ly6G+ CD11b+ neutrophils	Chil3, Camp, Lcn2, Ltf, Mmp9, Csf3r, IL1β, Ccl6
Alshetaiwi et al., 2020	GSE139125	Mouse	Breast	PT	Polyomavirus middle T oncoprotein breast cancer	FACS purification of live CD45+CD11b+Gr1+ cells	Ly6g, Cxcr2, Camp, Arg2, Cebpe, Retnig, Tuba1b, Cdc20
Azizi et al., 2018	GSE114727	Human	Breast	PT	Breast carcinomas and adjacent healthy tissue	PhenoGraph clustering; genome-wide correlations between cluster mean expression and previously characterized transcriptional profiles of sorted immune cells	CXCR1, MME, CXCR2, CSF3R, IL-1βR2, IFITM2, S100A8, S100A12, ICAM3, CXCL1
Wu et al., 2022	OEP001756	Human	Liver	MET	Colorectal cancer liver metastasis	Tissue dissociation and reservation of CD45^+^ clusters at single-cell resolution	LYZ, FCGR3B
Zhang et al., 2020	GSE146771	Human	Colon	PT	Colorectal cancer	Neutrophils were not sufficiently captured	N/A

Abbreviations: MET = metastatic tumor, N/A = unavailable, PT = primary tumor.

^a^Neutrophil markers used in the original publications to identify neutrophils.

**TABLE 2:** Links to software processing pipelines on Github

Software	Github Identifier
Seurat	https://github.com/satijalab/seurat
Slingshot	https://github.com/kstreet13/slingshot
TradeSeq	https://github.com/statOmics/tradeSeq
ClusterProfiler	https://github.com/YuLab-SMU/clusterProfiler
EnrichR	https://github.com/wjawaid/enrichR
CellChat	https://github.com/sqjin/CellChat
Cellranger	https://github.com/10XGenomics/cellranger
CellTypist	https://github.com/Teichlab/celltypist
tophat2	https://github.com/DaehwanKimLab/tophat2
Bowtie	https://github.com/BenLangmead/bowtie
HTSeq	https://github.com/simon-anders/htseq
DESeq2	https://github.com/mikelove/DESeq2

### Mouse Housing and Ethics

All animal experiments were performed in accordance with the UK Home Office project licenses 70/9112 and PP3908577 and were reviewed and approved by the University of Glasgow Animal Welfare and Ethical Review Board. Mice were fed standard chow diet and given drinking water *ad libitum*. A mixture of individually ventilated cages and conventional open top cages were used. Both sexes of mice were used in ageing models, with male mice used for transplants. Supplementary Table S1 summarizes the numbers, sex, and genotype of the mice used in this study.

### In-House Generated Mouse Models for scRNA-seq

The different intestinal cancer models are listed in Table 3. Two models of tumorigenesis were used: aged genetically engineered mice; and intracolonic transplants of murine-derived organoids (Supplementary Table S1). All genetically engineered mouse models (GEMM) were induced with a single 2 mg intraperitoneal injection of tamoxifen (Sigma-Aldrich T5648) and aged to a clinical endpoint. For transplant mice, murine tumor-derived organoids were injected intracolonically into male immune competent C57BL/6J mice (Charles River strain 632) using previously described methods (24). Tumor organoids were mechanically dissociated into fragments by pipetting and washed twice in PBS. Each mouse was injected with the equivalent of one well of a 6-well plate in 70 μL of PBS. This was injected into the colonic submucosa using a Karl Storz TELE PACK VET X LED endoscopic video unit with associated needle.

**TABLE 3:** Description of colorectal cancer mouse models used

Mouse model	Mutations	Reference
AKPT Transplant	villin-Cre^ERT2^*Apc*^fl/fl^*Kras*^G12D/+^*Trp53*^fl/fl^*Alk5*^fl/fl^	(58)
BP GEMM	villin-Cre^ERT2^*Braf*^V600E/+^*Trp53*^fl/fl^	(4, 59)
BPN GEMM	villin-Cre^ERT2^*Braf*^V600E/+^*Trp53*^fl/fl^*Rosa26*^N1icd/+^	(59)
KP GEMM	villin-Cre^ERT2^*Kras*^G12D/+^*Trp53*^fl/fl^	(5)
KPN GEMM and Transplant	villin-Cre^ERT2^*Kras*^G12D/+^, *Trp53*^fl/fl^, *Rosa26*^N1icd/+^	(5)

### Tissue Processing

PTs were excised into PBS on ice. The tumor was then chopped into a smooth paste using a McIlwain Tissue Chopper. The paste was transferred to GentleMACS C tubes (Miltenyi Biotec, 130-093-237) with digestion enzymes from the Miltenyi Mouse Tumor Dissociation Kit (Miltenyi Biotec, 130-096-730; 2.35 mL of RPMI1640, 100 μL Enzyme D, 50 μL Enzyme R, and 12.5 μL Enzyme A). Samples were run on a GentleMACS Octo Dissociator with Heaters (Miltenyi Biotec, 130-096-427) using the 37C_m_TDK_1 programme. After digestion, samples were briefly spun, 10 mL of RPMI-10%FBS-2 mmol/L Ethylenediaminetetraacetic acid (EDTA) was added and passed through a 70 μm strainer. The resultant suspension was then spun down at 900 RPM for 2 minutes at 4°C, supernatant discarded, and the pellet resuspended in 0.5 mL DPBS+0.05% BSA and transferred to a FACS collection tube on ice.

### scRNA-seq

Dissociated cells were sorted using a BD FACSAria (BD Biosciences) and DAPI (Invitrogen, D1306) to remove dead cells, then loaded onto a Chromium Chip G using reagents from the 10x Chromium Single-Cell 32 v3 Gel Bead Kit and Library (10x Genomics) according to the manufacturer's protocol. Libraries were analyzed using the Bioanalyzer High Sensitivity DNA Kit (Agilent Technologies) and sequenced on the Illumina NovaSeq 6000 with paired-end 150-base reads. Sequence alignment of single-cell data to the mm10 genome was performed using the count tool from Cellranger (version 6.1.2) according to the developers' instructions, generating barcodes, features, and matrix output files for each sample. Subsequent analysis was done in R (version 4.1.1) using Seurat (version 4.0.4). Samples were input using the Read10X function, filtered to include cells with a minimum of 100 expressed genes and genes that are present in at least three cells, then further filtered to only include cells with <5% mitochondrial genes, <10% hemoglobin genes, >100 genes/cell and >400 reads/cell. Samples were then integrated by RPCA using the IntegrateData function before being scaled and normalized. Dimension reduction was performed using PCA followed by clustering using the FindNeighbors and FindClusters functions. Marker genes for individual clusters were determined using the FindAllMarkers function. Cell types were annotated using CellTypist and custom gene lists, and subset using the subset function.

### Bulk-RNA-seq of Autochthonous Colorectal Cancer Mouse Models

Tissue processing, RNA isolation and sequencing were performed as described previously (5). Briefly, PTs were harvested from the intestine of 4 villin-Cre^ERT2^*Kras*^G12D/+^*Trp53*^fl/fl^*Rosa26*^N1CD/+^ (KPN) mice (model of metastatic colorectal cancer), and of these mice, 2 also had liver metastases which were harvested (one mouse had multiple liver metastases harvested). Liver tissue was harvested from 5 wild-type (WT) mice on a C57BL/6 background. Tumors from intestine, liver metastases, and liver were processed using the Mouse Tumor Dissociation Kit (Miltenyi Biotec #130-096-730) as per the manufacturer's instructions, along with blood obtained by cardiac puncture upon terminal anesthesia. Neutrophils were sorted on the basis of CD48^–/lo^Ly6G^+^, CD11b^+^Ly6G^+^ expression and RNA was extracted using the RNeasy Mini kit (QIAGEN, #74104). Purified RNA quality was tested on an Agilent 2200 Tapestation using RNA screen tape. Libraries for cluster generation and RNA sequencing were prepared using the Illumina TruSeq RNA LT Kit after assessing RNA quality and quantity on an Agilent 2200 Tapestation (D1000 screentape) and Qubit (Thermo Fisher Scientific), respectively. Libraries were run on an Illumina NextSeq 500 using the High Output 75 cycles kit. Quality checks on the raw RNA-seq data files were done using fastqc and fastq_screen (versions 0.11.2 and 0.11.3, respectively). RNA-seq paired-end reads were aligned to the GRCh38 mouse genome using tophat2 with Bowtie (versions 2.0.13 and 2.2.4.0, respectively). Expression levels were determined and analyzed using HTSeq (version 0.6.1) in R (version 3.2.2), utilizing Bioconductor data analysis suite and DESeq2.

### IHC of Human Colorectalcancer Liver Metastasis (CRCLM) Tissue

Studies were conducted in line with the Declaration of Helsinki and all patients provided written informed consent for their tissues to be used for research purposes. Application to access patient tissue was authorized by the NHS Greater Glasgow and Clyde Biorepository under their NHS Research Ethics Committee approval with ethical approval granted in biorepository application no. 602. Upon successful metastatic liver resections, surplus tissue was stored in 4% paraformaldehyde at 4°C for 20–48 hours. Samples were then transferred to 70% ethanol and processed by standard histology processing techniques.

The following antibodies were stained on a Leica Bond Rx autostainer: CD3 (ab16669, Abcam) and TXNIP (40-3700, Thermo Fisher Scientific). All formalin-fixed paraffin-embedded (FFPE) sections underwent on-board dewaxing (AR9222, Leica) and epitope retrieval using ER2 retrieval solution (AR9640, Leica) for 20 minutes at 95°C. Sections were rinsed with Leica wash buffer (AR9590, Leica) and peroxidase block was performed (Intense R kit; DS9263, Leica) for 5 minutes. Primary antibodies were added at optimal dilutions (CD3, 1/100; TXNIP, 1/400) then rabbit envision secondary antibody (K4003, Agilent) was applied for 30 minutes. Sections were rinsed and visualized using 3,3′-Diaminobenzidine (DAB) in Intense R kit.

FFPE sections for CD11b/ITGAM (49420, Cell Signaling Technology) staining were loaded into the Agilent pretreatment module for dewaxing and heat-induced epitope retrieval using high pH target retrieval solution (TRS; K8004, Agilent). Sections were heated to 97°C for 20 minutes in high pH TRS buffer, rinsed in flex wash buffer (K8007, Agilent) then loaded onto the Dako autostainer. Peroxidase blocking (S2023, Agilent) was performed for 5 minutes. Primary CD11b/ITGAM antibody was added (1/400) for 35 minutes, then rabbit envision secondary antibody was applied for 30 minutes. Sections were rinsed before applying Liquid DAB (K3468, Agilent) for 10 minutes. Sections were washed in water and counterstained with hematoxylin Z (RBA-4201-00A, CellPath). Finally, all sections were rinsed in tap water, dehydrated through graded ethanol's, placed in xylene then coverslipped using DPX mountant (SEA-1300-00A, CellPath).

### Organoid Culture

Tumoroids derived from KPN mice were used as described previously (5). Briefly, advanced DMEM/F12 was supplemented with 10 mmol/L HEPES (15630080), 2 mmol/L l-glutamine (25030081), 100 U/mL/100 μg/mL penicillin/streptomycin (15140122), N2-supplement (17502001), and B27 supplement (17504044; all from Gibco or Thermo Fisher Scientific) and is referred to as ADF base from hereon. EN medium was prepared by supplementing ADF base with 100 ng/mL Recombinant Murine Noggin (250–38) and 50 ng/mL recombinant human EGF (AF-100-15; both from Peprotech). Organoids were cultured in Matrigel and EN medium in 5% CO_2_ at 37°C, with passaging every 2–3 days.

### Intrasplenic Injection of Tumor Cells

Following culture of KPN organoids as described above, tumor organoids were released from plating by vigorous scraping followed by washing in cold PBS. Organoids were fragmented with vigorous pipetting, followed by incubation for 7 minutes at 37°C in 0.25% trypsin in EDTA-PBS. Following quenching of trypsinization by immersion in 10% FBS, cells were passed through a 40 µm strainer, counted using the Countess Automated Cell Counter (Thermo Fisher Scientific, AMQAF2000), and resuspended in PBS to achieve a final volume of 1 × 10^7^ cells/mL. Mice were then injected with 5 × 10^5^ cells intrasplenically in a volume of 50 µL.

### Mouse Models for Functional Analysis

GEMMs were selectively bred in house using the *Cre-*lox system with mice with an *Mrp8-Cre* (25) crossed with mice carrying a conditional deletion of *Cxcr2* (26) on an *n* ≥ 9 C57BL/6 background and genotyped at Transnetyx. All mice in these studies were homozygous for conditional deletion of *Cxcr2* (*Cxcr2*^fl/fl^), with Mrp8-Cre deficient mice (*Mrp8*CreN) used in control, and Mrp8-Cre proficient mice (*Mrp8*CreYCxcr2Δneut) used in experimental groups.

Where indicated in the experimental design, samples were either taken from healthy male and female mice ages between 14 and 20 weeks, or from mice ages between 17 and 21 weeks that were injected intrasplenically with 500,000 single cells prepared from KPN organoids as described above. All mice were sampled at 28 days following injection of tumor cells. No mice were excluded from analysis. No formal randomization of mice was carried out, allocation of mice to groups was unblinded, but surgery and data analysis were blinded.

### Tissue Preparation for Sorting

Whole livers from healthy mice, and those 28 days after KPN intrasplenic injection were fragmented and digested using the Mouse Tumor Dissociation Kit (Miltenyi Biotec, 130-096-730) in GentleMACS tubes on the GentleMACS Octo dissociator with heaters (Miltenyi Biotec, 130-096-427) on the setting 37C_m_LIDK_1. Cells were passed through a 40 µm strainer and enzymatic digestion was quenched using RPMI1640 10% FBS 2 mmol/L EDTA. Cells were then processed using debris removal solution (Miltenyi Biotec, 130-109-398). Blood was suspended in erythrocyte lysis buffer on ice, and then passed through a 70 µm strainer following quenching in RPMI1640 10% FBS 2 mmol/L EDTA. Liver and blood samples were then resuspended in PBS. T cells for coculture were obtained from WT mice. Spleen tissue was passed through a 70 µm filter and washed through with RPMI1640 10% FBS 2 mmol/L EDTA. Following centrifuge, spleen cells were resuspended in PBS.

### Flow Cytometric Sorting of Neutrophils

Neutrophils for coculture with KPN organoids were positively selected using FACS. Liver and blood samples prepared as described above were stained with TruStain FcX (anti-mouse CD16/32) Antibody (BioLegend, 101319) at a 1:200 concentration in 300 µL PBS 1% BSA for 10 minutes on ice in the dark. Subsequently, 300 µL of antibody cocktail with the following antibodies was added: CD45-SB600 (Thermo Fisher Scientific, 63-0451-82), Ly6G-APC (BioLegend, 127614), CD11b-FITC (BioLegend, 553310), CD48-PE/Cy7 (BioLegend, 103424), and cells were incubated for 30 minutes in the dark on ice. Gating of neutrophils was unaffected by expression of Mrp8Cre-GFP at 525/50 nm, as with the excitation peak of CD11b-FITC. Cells were then washed and resuspended in PBS 1% BSA, and 1 µL of 1 mg/mL DAPI (Thermo Fisher Scientific 62248) was added.

On the BD FACSAria III (BD Biosciences) neutrophils were sorted as Live, CD45^+^, CD48^–^/lo, CD11b+, Ly6G+, and collected in PBS 50% FBS.

### Negative Selection of Neutrophils and T-cell Coculture

Neutrophils for coculture with T cells, from healthy mouse liver and blood as well as liver and blood from mice 28 days after KPN intrasplenic injection were negatively selected using MojoSort Mouse Neutrophil Isolation Kit (BioLegend, 480058) and their described protocol. T cells for the same experiments were isolated from WT mouse spleen tissue using the MojoSort Mouse CD3 T cell isolation kit (BioLegend, 480024) and their described protocol. Cells were incubated in MojoSort buffer 1x (BioLegend, 480017) with 10 µL of biotin-antibody cocktail per 1 × 10^7^ cells. Following incubation on ice for 15 minutes, 10 µL of streptavidin nanobeads were added, and further incubated on ice for 15 minutes. Samples were then magnetically sorted, with the remaining suspension containing the cells of interest. The remaining cells that had been magnetically trapped were then resuspended in MojoSort buffer and once again magnetically sorted to increase yield.

Following counting, T cells were stained with CellTrace Yellow Proliferation kit (Thermo Fisher Scientific, C34567). A total of 500,000 T cells were placed into a well of a flat-bottomed 96-well plate, and cocultured with 1 × 10^6^ neutrophils in 250 µL Iscove's modified Dulbecco's medium, 10% FBS, 50 mmol/L 2-mercaptoethanol with 2 mmol/L l-glutamine and 100 U/mL/100 µg/mL penicillin-streptomycin. Cells were harvested following 44 hours of incubation at 37°C 21%O_2_ 5% CO_2_.

### Neutrophil-KPN Organoid Cocultures

KPN organoids were propagated in culture as described above. Three days prior to collection of neutrophils, 50,000 KPN organoid cells were seeded in each well per experimental condition. Following positive selection of neutrophils using FACS as described above, 50,000 neutrophils were placed in coculture with KPN organoids grown from previously seeded cells. Neutrophils and KPN organoids were suspended in Matrigel and plated in a flat-bottomed 24-well plate, in EN media. These were incubated at 37°C 21%O_2_ 5% CO_2_ for 12 hours.

### Assessment of Extracellular DNA Release

To quantify extracellular DNA release, just prior to coculture, neutrophils were stained with 1 µmol/L cell tracker red (Thermo Fisher Scientific, C34552) in ADF base, and incubated in this for 45 minutes at 37°C. Sytox Green nucleic acid stain (Thermo Fisher Scientific, S7020) was added to EN medium in which the neutrophils and KPN organoids were cocultured.

A Nikon TE2000 microscope was used to obtain time-lapse images every 30 minutes for 11 hours, with three technical replicates per condition. Brightfield (organoid imaging), 525/50 nm detecting Sytox green (excitation max 504 nm, emission max 523 nm) fluorescence for release of DNA and 630/75 nm detecting neutrophils stained with cell tracker red (excitation max 577 nm, emission max 602 nm) fields were captured at each timepoint. Images were processed using FIJI, and mean fluorescence at each timepoint was measured and normalized against KPN organoid-only controls. Statistical analysis was performed in GraphPad Prism (Dotmatics), using a Mann–Whitney test at each timepoint to test for statistical significance.

### Flow Cytometric Analysis

Three flow cytometry panels were designed. The first, referred to as the T-cell proliferation panel, contained CD8a-BV421 (BioLegend, 100753), CD4-BUV395 (BD Biosciences, 563790), and CD3-PE/Cy7 (BioLegend, 100220). The second, referred to as the organoid viability panel contained Annexin V-FITC (BioLegend, 640906), EpCAM-BV650 (BioLegend, 118241), propidium iodide (PI; BioLegend, 421301), and CD45-PE (BioLegend, 103106). The third, referred to as the neutrophil activation panel, contained MPO-FITC (Cambridge Bioscience, HM1051F-100UG), MMP9-AF405 (Biotechne, NBP2-59699AF4050), CD62L-BUV395 (BD Biosciences, 740218), CD45-SB600, CD48-BV711 (BioLegend, 103439), CD11b-BUV805 (BD Biosciences, 741934), Ly6G-BV510 (BioLegend, 127633), and LTF-AF680 (Stratech, BS-5810R-A680-BSS).

Neutrophils from blood and liver of mice 28 days following intrasplenic injection of KPN tumor cells and T cells from healthy WT mice (*n* = 14) in coculture were harvested and incubated for 20 minutes on ice in the dark with 100 µL LIVE/DEAD fixable near-IR stain kit (Thermo Fisher Scientific L10119) at 1:1,000 dilution in PBS. Following washing in PBS 1% BSA, cells were resuspended in 25 µL of PBS 1% BSA containing a 1:200 dilution of TruStain FcX (anti-mouse CD16/32) Antibody and incubated on ice in the dark for 20 minutes. The T-cell proliferation panel antibody cocktail was then added in 25 µL PBS 1% BSA and incubated for 30 minutes in the dark on ice. Following washing and resuspending cells in PBS, they were fixed in PBS 4% paraformaldehyde for 15 minutes at room temperature. Cells were then washed and resuspended in PBS.

Neutrophils in coculture with KPN organoids were all from healthy mice. These were harvested by incubating at 37°C in 200 µL TrypLE Express Enzyme (Thermo Fisher Scientific, 12604013), and then stained and fixed as described above, using the organoid viability panel and the neutrophil activation panel in place of the T-cell proliferation panel. Following fixation, samples stained with the neutrophil activation panel were resuspended in 1x permeabilzation buffer (Thermo Fisher Scientific, 00-8333-56) containing MPO-FITC, MMP9-AF405, and LTF-AF680 antibodies. Cells were then washed and resuspended in PBS for analysis on the flow cytometer.

All cells were initially identified for analysis by gating on FSC-A and SSC-A, and subsequently on FSC-A and FSC-H. Cells were then gated on LIVE/DEAD NIR. T cells were defined subsequently as CD3^+^, and CD4^+^ and CD8^+^ subsets. CellTrace Yellow was used to analyze T-cell proliferation, with defined generations of T cells based on strength of the fluorescent signal. Generation 0 are undivided T cells, generation 1 have undergone one division, generation 2 two divisions etc. Neutrophils were defined as CD45^+^, CD48^–^/lo, CD11b^+^, Ly6G+, and MPO, MMP9, LTF and CD62 L used as markers of activation. Tumor cells were defined as CD45–, EpCAM+, with Annexin V+/PI–, PI+/Annexin V–, and Annexin V+/PI+. All Flow analysis was performed in FlowJo software (BD Life Sciences), and statistical analysis was performed in GraphPad Prism, using unpaired *t* tests to establish statistical significance in T-cell proliferation assays and two-way ANOVA in neutrophil activation, maturity, and viability.

### Quantification of Neutrophil Response to Exogenous Stimuli

Neutrophils from 6 BALB/c WT mice were flushed from the bone marrow following lethal injection of phenobarbital. Neutrophils were negatively selected from the bone marrow solution using the MojoSort Mouse Neutrophil Isolation Kit as described above. Neutrophils were then plated with either vehicle, 20 ng/mL GMCSF (Peprotech, 315-03-20ug) or 10 ng/mL IL1β (ImmunoTools, 12340013) and cultured for 2 hours at 37°C in 5% CO_2_ and 21% O_2_. Neutrophils from 3 mice were cultured with vehicle or GMCSF, and with vehicle or IL1β from the remaining 3 mice. As all mice were WT, they were not randomized, but analysis of results was blinded. One plate was excluded from analysis (neutrophils treated with IL1β for analysis of Cxcr2 due to issues with sealing the plate. Cells were collected into TRIzol (Life Tech), and RNA prepped as per manufacturer's instructions. cDNA was synthesized using 1 µg RNA as template using Quantitect Reverse Transcription Kit (Qiagen) as per the manufacturer's instructions. qPCR was performed using x1 SyBr green (Qiagen), 1:10 dilution of cDNA template, 0.5 µmol/L forward primer (custom made, Life Tech), 0.5 µmol/L reverse primer (custom made, Life Tech), and reactions conducted using a three-step protocol, corresponding to 95°C for 3 minutes, x40 cycles of 95°C for 20 seconds, 60°C for 20 seconds, 72°C for 20 seconds, with a final extension of 72°C for 5 minutes, and melt-curve from 65°C to 95°C in 0.5°C increments. Data were collected and analyzed in CFX Manager Software (Bio-Rad). Expression of genes of interest was normalized to Actb. Statistical significance was tested using an unpaired *t* test. Primer sequences can be found in Supplementary Table S2.

### Data Availability

All relevant code has been deposited on Github (https://github.com/ranafetit/NeutrophilCharacterisation). Data analyzed from publicly available datasets were obtained from GEO database and National Omics Encyclopaedia as listed in Table 1. Data generated independently from GEM and transplant models of colorectal cancer in this study are available from Zenodo at (https://doi.org/10.5281/zenodo.8133995). Any other data generated in this study are available within the article and its Supplementary Data, or upon request from the corresponding author.

## Results

### Neutrophils Exhibit Distinct Tissue-specific and Tumor-specific Signatures

**FIGURE 1:**
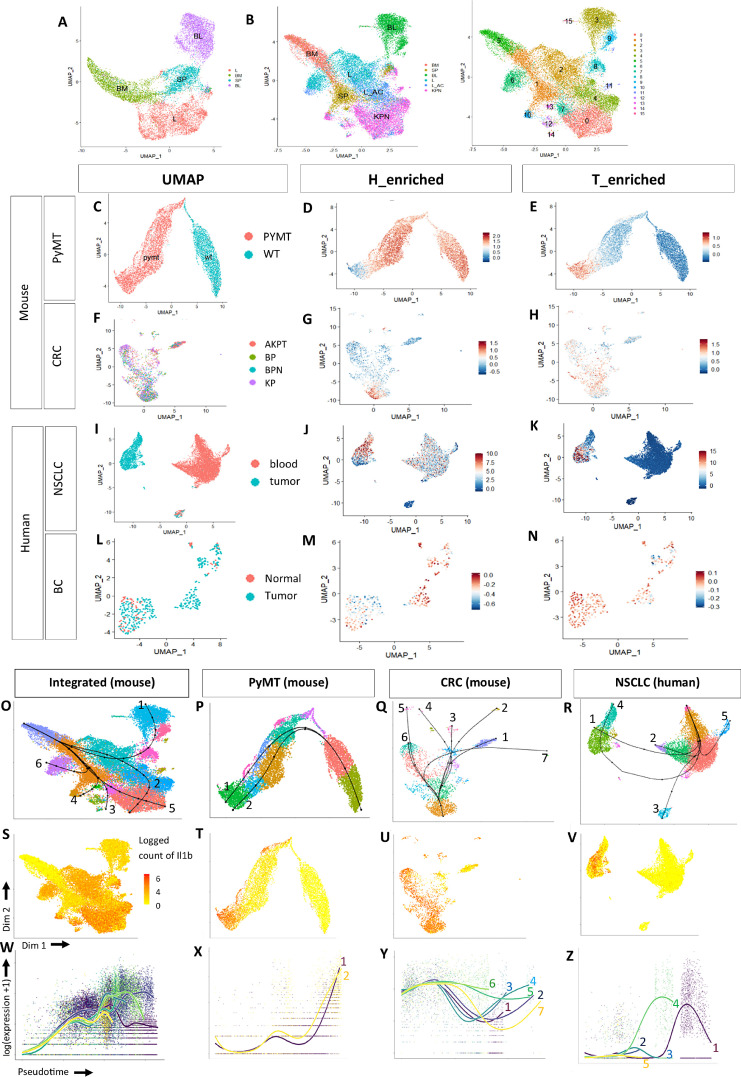
Characterization of neutrophil signatures and lineages in health and PTs. **A,** Uniform Manifold Approximation and Projection for Dimension (UMAP) plot of healthy neutrophils grouped by tissue type. BM: healthy bone marrow, SP: healthy spleen, L: healthy lung, BL: healthy blood. **B,** UMAP plots of healthy and tumor-derived neutrophils grouped by tissue type and Seurat clusters (0–15). BM: healthy bone marrow, SP: healthy spleen, L: healthy lung, BL: healthy blood, L_AC: lung adenocarcinoma, KPN: colorectal cancer with *Kras*, *Trp53* and Notch mutations. **C,** UMAP plot of neutrophils in mouse breast cancer model. WT: healthy breast tissue, PyMT: polyomavirus middle-T oncoprotein tumor. **D** and **E,** Scoring of H_enriched and T_enriched neutrophil signatures. **F,** UMAP plot of neutrophils in mouse colorectal cancer model. All neutrophils are tumor-derived. **G** and **H,** H_enriched and T_enriched signatures in colorectal cancer (CRC). **I,** UMAP plot of human NSCLC neutrophils. Blood: blood-derived, tumor: tumor-derived. **J** and **K,** H_enriched and T_enriched signatures NSCLC. **L,** UMAP plot of human breast carcinoma (BC) neutrophils. Most neutrophils are tumor-derived. **M** and **N,** H_enriched and T_enriched signatures in breast carcinoma. **O–R,** Unsupervised pseudotime analysis of neutrophils in mouse and human datasets. Lineages in the individual datasets are numbered. **S–V,***IL1β/IL1β* is differentially expressed at the end of tumor-specific lineages. **W–Z,** Estimated smoothers for *IL1β/IL1β* expression over pseudotime across the different lineages.

To examine the transcriptomic signatures of neutrophil subtypes in healthy and tumor tissue, we integrated neutrophil clusters from bone marrow, blood, lung, and spleen of healthy mice (16), together with neutrophils from tumor-bearing mouse models of non–small cell lung cancer (NSCLC; ref. 15) and colorectal cancer (KPN; Fig. 1). Both NSCLC and colorectal cancer tumor models shared a comparable C57BL/6 background with *Kras* and *Trp53* mutations. For colorectal cancer, neutrophils were derived from two models of tumor genesis: aged GE mice and intracolonic transplants of murine derived organoids, with the majority being from the latter (Supplementary Fig. S1A). Unsupervised clustering of neutrophil transcriptomic signatures revealed distinct neutrophil clusters based on their tissue of origin in health (Fig. 1A). KPN and lung adenocarcinoma neutrophils formed distinct tumor-specific clusters, suggesting transcriptomic differences between neutrophils in KPN, lung adenocarcinoma and healthy tissues (Fig. 1B).

KPN neutrophils encompassed clusters: 0, 4, 7, 8, 10, 11, 12, and 14 (Fig. 1B; Supplementary Fig. S1B and S2A). Clusters 0 and 7 were enriched for *Cxcl2* and *Thbs1*, which encode proteins that influence neutrophil motility and chemotaxis. Cluster 0 also expressed *Ccl4* and *Ccl3*, critical for T-cell recruitment and antitumor immunity (ref. 27; Supplementary Table S3). Cluster 10 expressed *Cd74*, which plays a role in neutrophil accumulation (28). Cluster 4 was common to both KPN and NSCLC PTs (Fig. 1B; Supplementary Fig. S1B), and expressed *Cdkn1a*, *Ppia*, *Gngt2*, *Ier3*, and *Rps27l* (Supplementary Table S3). Three smaller clusters were shared between both PTs: Cluster 12 was enriched for the lysosomal genes *Lyz1* and *Psap*, Cluster 14 expressed *Ppia*, *Jun*, and *Slfn4*, and Cluster 11 was enriched for *S100a10* and *Ptma*. Finally, Cluster 8 was equally conserved across healthy and tumor-associated neutrophils (Fig. 1A and B; Supplementary Fig. S1B) and was enriched for IFN markers: *Isg15*, *Rsad2*, *Ifit3*, *Ifit1*, and *Slfn4* (Supplementary Table S3), a phenomenon previously reported in several scRNA-seq studies of neutrophils (29).

### Neutrophils in PTs Encompass Two Transcriptional Subtypes in Mice

Using the highly expressed markers in healthy and tumor tissues (Supplementary Table S3), we defined two neutrophil signatures. The first represented neutrophils associated with healthy tissue (Healthy-enriched; H_enriched). This subtype was observed in both healthy and tumor tissue. The second signature was specific to tumor-associated neutrophils (Tumor-enriched; T_enriched; Fig. 1C–N).

To validate the established signatures across different tissues and tumors, we scored them on additional neutrophil scRNA-seq datasets derived from a mouse model of healthy and breast cancer tissue, utilizing the mouse mammary tumor virus promoter–driven expression of the polyomavirus middle-T oncoprotein (PyMT, GSE139125) and neutrophils derived from a compendium of colorectal cancer mouse models generated in our lab (Table 2).

In PyMT, neutrophils clearly separate into distinct healthy and tumor-specific clusters, recapitulating the findings in NSCLC and colorectal cancer datasets (Fig. 1C and F). Signature scoring in both PyMT and colorectal cancer mouse models revealed that within the PT, tumor-specific neutrophils can be separated into two subgroups: (i) activated neutrophils, which are transcriptionally similar to neutrophils from healthy tissue (Fig. 1D and G), and (ii) a subtype specific to PTs (Fig. 1E and H). Both signatures were preserved in both GEMM and transplant models of colorectal cancer (Supplementary Fig. S2B and S2C for healthy and tumor derived neutrophils, respectively).

### Neutrophil Signatures are Conserved Between Mouse and Human

We then investigated whether these signatures (Table 4) could be translated to humans, using two datasets: patient-derived neutrophils from NSCLC tumor and blood (Fig. 1I, GSE127465); and breast carcinomas and adjacent healthy tissue (Fig. 1L, GSE114727). Signature scoring in NSCLC confirmed the presence of both neutrophil subsets within the PT (Fig. 1J and K) recapitulating the trends observed in mice. Blood-derived neutrophils largely resemble the H_enriched subtype (Fig. 1J). Although the breast carcinoma dataset contained very few cells, we successfully observed the enrichment of both neutrophil subtypes in tumor-derived neutrophils (Fig. 1M and N). Our analysis validates the presence of both neutrophil transcriptomic subtypes in patient PTs, albeit to different extents in the different cancer types and tissues, implying a role of the neutrophil's environment in shaping their transcriptome.

**TABLE 4:** Healthy- and Tumor-enriched neutrophil signatures in human and mouse

Human			Mouse		
Healthy-enriched	Tumor-enriched	Metastasis-enriched	Healthy-enriched	Tumor-enriched	Metastasis-enriched
MMP8	CDKN1A	TXNIP	Mmp8	Cdkn1a	Txnip
IFITM2	PPIA	RIPOR2	Ifitm6	Ppia	Ripor2
IFITM3	IFITM1	CXCR2	S100a6	Ifitm1	Cxcr2
S100A6	TAGLN2	FKBP5	Lyz1	Tagln2	Fkbp5
LYZ	ISG15	CEBPD	Lyz2	Isg15	Cebpd
CTLA4	GNGT2	STK17B	Ctla2a	Gngt2	Stk17b
CHI3L1	CXCL8	SMAP2	Chil3	Cxcl12	Smap2
G0S2	CCL4	CTSS	G0s2	Ccl4	Ctss
FPR2	CD14	JAML	Fpr2	Cd14	Jaml
	RPS27L	IGHM		Rps27l	Ighm
	IER3	CD74		Ier3	Cd74
	CCL3			Ccl3	
	IFIT3			Ifit3	
	IFIT1			Ifit1	
	IL1’			IL1β	
	WFDC1			Wfdc17	
	THBS1			Thbs1	
	PTMA			Ptma	

### Pseudotime Analysis Demonstrates Neutrophil Lineages Progress from H_Enriched Toward T_Enriched Neutrophils

To investigate the developmental trajectory of neutrophils from health to cancer, we performed unsupervised pseudotime analysis on our integrated mouse dataset (Fig. 1O). Individual lineages are shown in Supplementary Table S4. Our trajectory analysis, which relies on cellular transcriptional information, recapitulated the neutrotime RNA velocity analysis (16), which relies on splicing dynamics. Lineage development is observed from bone marrow neutrophils to populations in the spleen and then blood in a stepwise transcriptomic progression in healthy neutrophil populations (Fig. 1O; Lineage 1, Supplementary Table S4). Additional lineages then become apparent as tumor-specific clusters develop. This trend was observed in both the murine PyMT and breast carcinoma datasets (Fig. 1P and Q). In the human NSCLC neutrophil dataset, lineages begin from the blood-derived clusters enriched for the H_enriched signature, and progress toward the tumor-derived clusters enriched for the T_enriched signature (Lineages 1 and 4, Supplementary Table S4; Fig. 1R). Our data identified a developmental trajectory beginning with activated, healthy neutrophils and ending at tumor-specific neutrophils in these datasets.

### *IL1β* is a Driver of T_Enriched Neutrophil Signature

We analyzed gene expression along the different trajectories to identify genes that drive neutrophil differentiation from health to cancer-associated lineages. Specifically, we investigated the genes differentially expressed at the end of the lineages compared with the start. In our integrated mouse dataset, *Il1b* was upregulated in the lineages ending with the tumor clusters (Fig. 1S and W). *Il1b* was also among the top 30 lineage-specific differentially expressed genes and was specific to the T_enriched neutrophil clusters PyMT (Fig. 1T and X; Supplementary Table S5). This was true for colorectal cancer (Fig. 1U and Y; Lineages 4 and 6; Supplementary Table S6). In human NSCLC, the same trend was observed in Lineages 1, 2, and 4 (Supplementary Table S7; Fig. 1V and Z). The human breast carcinoma dataset was too small to perform such an analysis. Taken together, our lineage-specific differential gene expression analyses implicate *IL1β* in the progression of neutrophils towards the T-enriched population in PT.

### Neutrophils in Colorectal Cancer LM Display Heterogeneous Transcriptional Programmes

**FIGURE 2:**
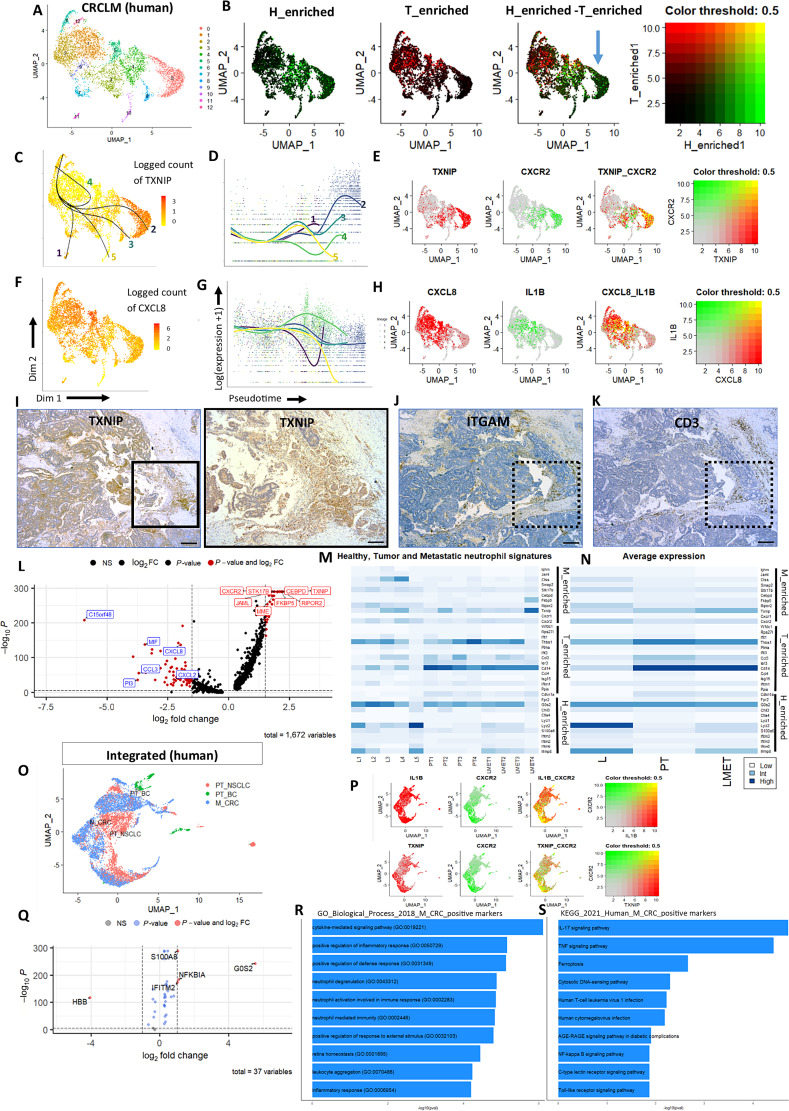
Characterization of neutrophils in metastasis. **A,** UMAP of neutrophils in CRCLM. **B,** Coexpression of H_enriched and T_enriched signatures. One cluster is not enriched for either signature (blue arrow). **C** and **D,** Unsupervised pseudotime analysis and estimated smoothers for TXNIP expression over the different numbered pseudotime lineages. **E,** Coexpression of TXNIP and CXCR2. **F** and **G,** Expression and estimated smoothers for CXCL8 over pseudotime. **H,** Coexpression of CXCL8 and IL1β. **I,** IHC staining of TXNIP in a patient CRCLM sample at 4x (left) and 10x (right). Scale bars = 50 µm. **J** and **K,** IHC staining of ITGAM (Neutrophils) and CD3 (T cells) in a patient CRCLM sample at 4x, scale bars = 50 µm. Dashed squares indicate regions where immune cells cluster. **L,** Differentially expressed genes in metastasis-specific neutrophil cluster. **M,** H_enriched, T_enriched, and M_enriched gene signatures in mouse bulk-RNA-seq neutrophil dataset. **L,** Healthy liver tissue, PT: Primary tumor, LMET: Liver metastasis. **N,** Averaged expression of the individual genes of the three signatures. **O,** UMAP plot of integrated human neutrophils from PT and metastatic (M) datasets of different cancers. **P,** Coexpression of *CXCR2* with *IL1β* (top) and *TXNIP* (bottom). **Q,** Differential gene expression between neutrophils in malignancy compared with PT. **R** and **S,** GO and KEGG analysis of M_CRC neutrophils.

To investigate whether these neutrophil subtypes are present in metastatic cancer, we isolated neutrophils from the publicly available CRCLM dataset (ref. 18; Fig. 2A) and scored them for the two signatures we established in PTs. Neutrophils in CRCLM expressed both H_and T_enriched signatures. However, a remarkable overlap between the two signatures was observed, with no clear separation between the two subtypes, reflecting their heterogeneity (Fig. 2B). Some clusters were not enriched for either the H_ or T_enriched neutrophil signature (Fig. 2B, blue arrow), suggesting the presence of an additional transcriptionally segregated neutrophil population, specific to metastatic colorectal cancer.

Unsupervised pseudotime analysis revealed five neutrophil lineages, starting from the clusters enriched for the T_enriched signature (Fig. 2C). All lineages shared the same sequence for the first six clusters and differed at their terminal clusters (Supplementary Table S4). We focused on lineages 2 and 4 because Lineage 2 progressed toward the cluster not enriched for either signature observed in PTs while Lineage 4 terminated with a cluster expressing both signatures observed in PTs (Supplementary Table S4). Collectively, our findings highlight neutrophil plasticity, suggesting that there is no fundamental pathway across all diseases but rather multiple developmental pathways. We specifically focused on the progressive transcriptomic development of neutrophil phenotype from healthy to tumor-specific signatures in PTs and finally, a metastatic-specific neutrophil subtype in colorectal cancer.

### Human CRCLM-specific Neutrophils Display T-cell Suppressive Markers

To characterize the transcriptomic signature of the metastatic-specific neutrophil population, we identified marker genes for the lineage endpoints. Lineage 2 endpoint was enriched for the mRNA encoding Trx-interacting protein (*TXNIP*, Fig. 2C and D), the upregulation of which inhibits TRX1 and restrains late T-cell expansion (30). This cluster was also enriched for the chemokine receptor CXCR2 (Fig. 2E), which is a commonly studied target in murine models of cancer influencing metastatic burden, suggesting these neutrophils identified are functionally relevant (31). The endpoint of Lineage 4 (Cluster 5; Supplementary Table S4) was enriched for the chemokine *CXCL8* (Fig. 2F and G), the major ligand for G-Protein coupled receptor CXCR2 and associated with immune suppression and tumor progression in this context (32). This cluster also highly expressed *IL1B* (Fig. 2H), supporting our hypothesis that *IL1β* is implicated in the progression of neutrophils toward malignancy-associated phenotypes.

We confirmed the expression of *TXNIP* in a patient CRCLM sample (Fig. 2I), in tumor regions where immune cells cluster (Fig. 2J and K; dashed squares). Moreover, among the 10 most highly expressed markers in this cluster, compared with other neutrophil clusters were the genes: *RIPOR2* and *STK17B*, which are important for naïve T-cell quiescence, survival, and activation (refs. 33, 34; Fig. 2L; Supplementary Table S8). Our findings suggest that the metastasis-specific neutrophil subtype transcribes genes that are T-cell suppressive.

### Murine Neutrophils Express a Metastasis-specific Signature in CRCLM Bulk-RNA-seq Dataset

We selected the top 11 highly expressed genes in the metastasis-specific neutrophil clusters, which we called Metastasis-enriched (M_enriched) signature (Table 3; Fig. 2L–N; Supplementary Table S8). These genes were not strongly expressed in the PT datasets, and those that expressed them showed very low score for the signature (Supplementary Fig. S3A). We then compared the established H_enriched, T_enriched, and M_enriched gene signatures in a bulk-RNA-seq dataset generated from neutrophils from an autochthonous KPN mouse model of colorectal cancer PT, LM, and healthy liver tissue (L) we generated (Fig. 2M). Higher expression of H_enriched genes was observed in the healthy liver tissue, compared with the PT and LM tissue (Fig. 2M). Neutrophils in both PT and LM tissue equally expressed the T_enriched signature, with a higher expression of genes such as *Thbs1*, *Ccl3* and *Cd14* in PTs (Fig. 2M). The average expression of the individual genes across the three signatures revealed a gradual increase in *Txnip* expression, with this expression being highest in LM tissue (Fig. 2N). This further validates the trends observed in our scRNA-seq analysis and demonstrates cross-species relevance.

### GSE Analysis Implicates IL17/CXCR2 Axis in Metastatic Neutrophil Populations

We integrated patient-derived neutrophil scRNA-seq signatures from lung and breast cancer PTs (PT_NSCLC and PT_BC), with neutrophils from CRCLM tissue (M_CRC) to identify differences in gene expression between PT tissue and metastatic samples (Fig. 2O). Coexpression analysis revealed that neutrophils from both PT and LM tissue expressed *IL1B*; however, *CXCR2* expression was largely specific to LM (Fig. 2P). Metastatic neutrophils coexpressed *CXCR2* and *TXNIP* (Fig. 2P), highlighting the specificity of these markers to neutrophils in CRCLM. Differential gene expression between neutrophils in PT and in CRCLM revealed the upregulation of the NETosis marker *G0S2* and *NFKB1A* (Fig. 2Q). Supplementary Table S9 shows the top 10 differentially expressed genes, grouped by tumor type. Using the differentially expressed genes in M_CRC neutrophils (Supplementary Table S10), GO analysis revealed an upregulation of cytokine-mediated signaling and positive regulation of inflammatory response (Fig. 2R). KEGG analysis revealed the upregulation of IL17, TNF, and NFκB signaling pathways (Fig. 2S). This supports the data implicating the IL17/CXCR2 axis in metastatic neutrophil populations (35).

### CRCLM-derived CD4^+^ T Cells Transcriptionally Diverge from their PT Counterparts

**FIGURE 3:**
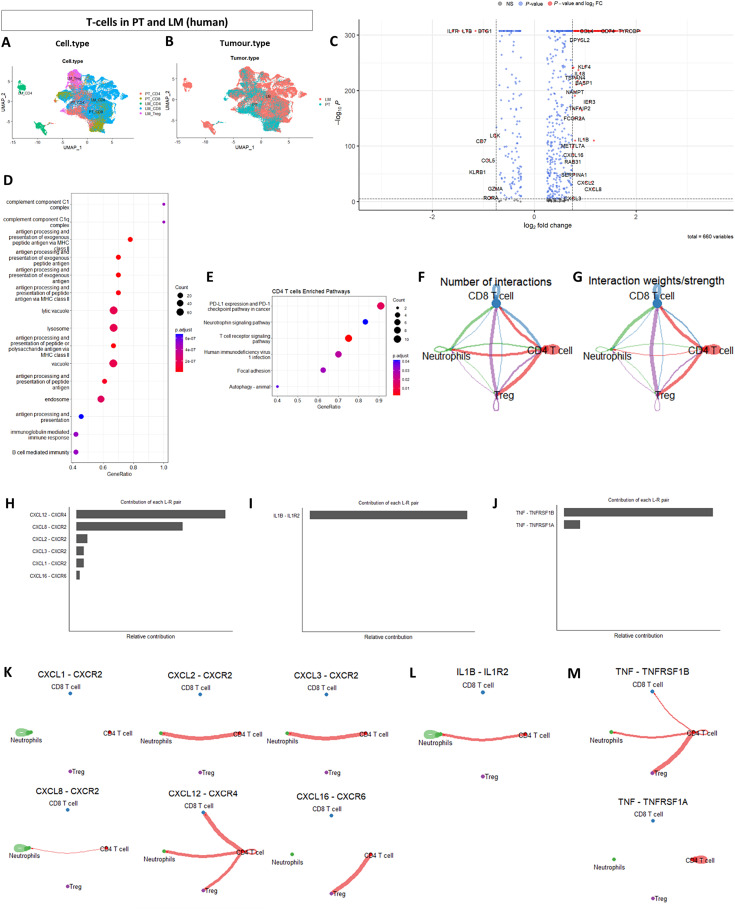
CD4^+^ T cells are transcriptomically altered in metastasis. **A** and **B,** UMAP plots of CD4^+^ and CD8^+^ T cells in PT and LM grouped by cell type and tumor type, respectively. **C,** Differential gene expression of CD4^+^ T cells in LM compared with PT. **D** and **E,** GO and KEGG analyses of metastatic CD4^+^ T cells. **F** and **G,** Global cell-cell communication network and the interaction strengths between neutrophils, CD4^+^, CD8^+^, and Tregs. **H–J,** The contribution of each L-R pair to the overall signaling pathway for CXCL, IL1, and TNF pathways. **K–M,** Visualization of the cell-cell communication patterns mediated by each significant L-R pair for the three pathways.

We revisited the publicly available datasets of colorectal cancer PT (19) and CRCLM (18) to isolate T cells and investigate transcriptomic differences in metastasis given the T-cell suppressive phenotype of neutrophils found in CRCLM (Fig. 3). Upon integration, CD8^+^ T cells from both PT and LM largely cocluster, reflecting their transcriptomic similarity. However, CRCLM-derived CD4^+^ T cells formed a distinct cluster (Fig. 3A and B). Differential gene expression analysis revealed 660 and 26 differentially expressed genes for the CRCLM-derived CD4^+^ and CD8^+^ T cells, respectively, compared with their equivalent PT populations (Supplementary Tables S11–S13). Henceforth, we focused on CD4^+^ T cells. *IL1B* and *CXCL8* were upregulated, the same genes we identified as drivers of metastatic neutrophil subtype (Fig. 3C), suggesting that the IL1β/CXCL8/CXCR2 axis drives the interaction between neutrophils and T cells in metastatic tissue.

In addition, we observed a downregulation of *RORA*, implicated in CD4^+^ T-cell activation (36), together with Granzyme A (*GZM*A) and proinflammatory lipid-mediator leukotriene B (*LTB*); further suggesting the possibility of impaired CD4^+^ T-cell function in CRCLM. GO and KEGG analyses revealed the dysregulation of biological processes converging on the complement system, antigen processing and presentation, together with perturbations in PD1-PD-L1 and T-cell receptor signaling pathways in the CRCLM-derived CD4^+^ T cells (Fig. 3D and E). Collectively, this suggests that the regulatory function of CD4^+^ T cells may be impaired in metastasis, contributing to an immunosuppressive phenotype.

### Neutrophils and CD4^+^ T Cells Interact Through IL1B, CXCL, and TNF Signaling Pathways in CRCLM

We then assessed the global cell-cell communication network using the Rpackage (CellChat) to investigate how the impaired CD4^+^ T cells in the metastatic niche may influence neutrophils and other T-cell subtypes. Signals from CD4^+^ T cells interact with CD8^+^ T cells and regulatory T cells (Treg) and, to a lesser extent, neutrophils (Fig. 3F and G). Outgoing signals from neutrophils are received by CD8^+^ T cells (Supplementary Fig. S3B), supporting our hypothesis that neutrophils impair CD8^+^ T-cell function in metastasis. Signals from CD8^+^ T cells are mostly autocrine and Tregs mainly influence CD8^+^ T cells (Supplementary Fig. S3B). We identified 20 signaling pathways showing significant communications between neutrophils, CD4^+^ T cells, CD8^+^ T cells, and Tregs (Supplementary Fig. S4). Supplementary Figure S5A–S5D show the significant L-R interactions between neutrophils, CD4^+^ T cells, CD8^+^ T cells, and Tregs to other target cell groups. Neutrophils primarily communicate with CD8^+^ T cells through the MHC-I pathway (Supplementary Fig. S5A). CD4^+^ T cells strongly interact with CD8^+^ T cells through MHC-I and MHC-II pathways and with Tregs through the MHC-II pathway (Supplementary Fig. S5B). CD8^+^ T cells communicate with CD4^+^ T cells and neutrophils through CD45 and ANNEXIN signaling pathways, respectively (Supplementary Fig. S5C). Finally, Tregs target CD8^+^ T cells through the MHC-I signaling pathway (Supplementary Fig. S5D).

We then focused on IL1β, CXCL, and TNF pathways, based on their involvement in defining the phenotype of metastatic neutrophils. CXCL12-CXCR4 and CXCL8-CXCR2 were the major L-R interactions observed where CD4^+^ T cells are major senders of the CXCL12-CXCR4 signals and CXCL8-CXCR2 signals are largely autocrine within neutrophils (Fig. 3H and K). IL1β-IL1βR2 significantly contributed to the IL1β pathway in CRCLM, an L-R interaction largely driven by the CD4^+^ T cells and neutrophil interactions, as well as neutrophils’ autocrine signaling (Fig. 3I and L). This observation supports our pseudotime findings in the different neutrophil populations. Finally, CD4^+^ T cells communicate with Tregs, neutrophils, and CD8^+^ T cells through TNF–TNFRSF1B interactions (Fig. 3J, M). Our findings suggest that within the metastatic niche, neutrophils primarily target CD8^+^ T cells through the MHC-I pathway, in addition to their autoregulation through CXCL and IL1β pathways with CD4^+^ T cells undertaking a prominent regulatory role.

### CD4^+^ T Cells are Dominant Signal Senders in CRCLM

**FIGURE 4:**
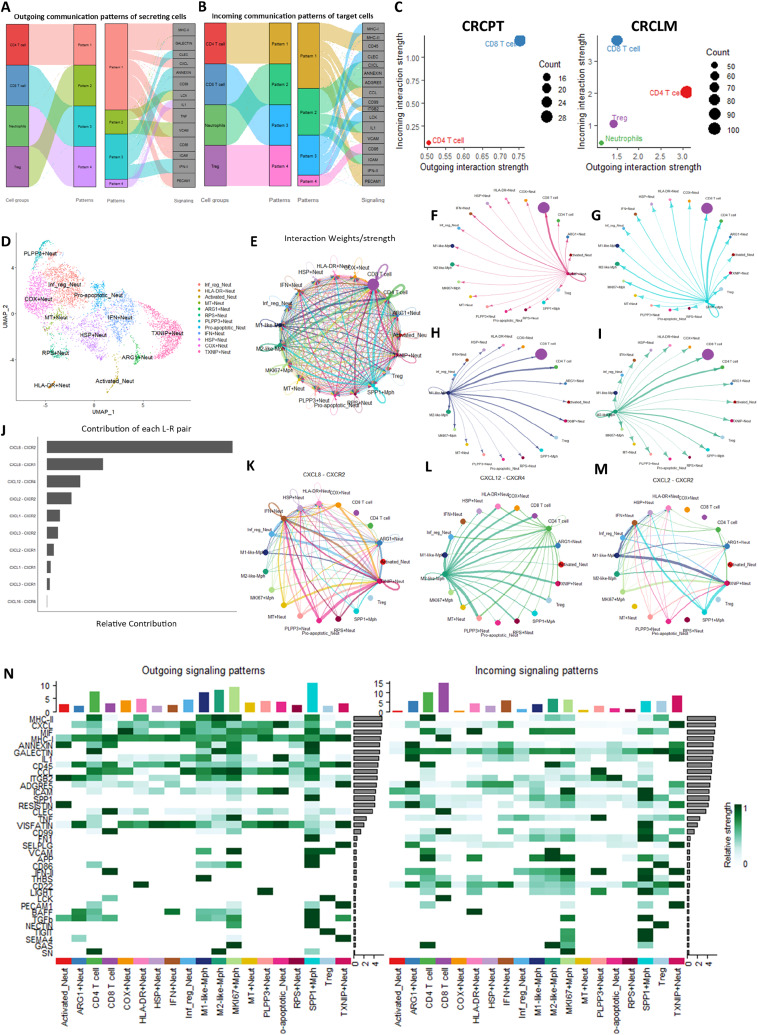
Signaling patterns in CRCLM. **A** and **B,** Outgoing and incoming signaling patterns in CRCLM. **C,** Cellular roles as dominant senders (sources) and receivers (targets) in CRCPT and LM. CRCPT dataset did not contain any Tregs or neutrophils. **D,** UMAP plot of neutrophil subtypes in CRCLM. **E,** Interaction strength of the global communication patterns between neutrophil, T-cell, and macrophage subtypes. **F–I,** Outgoing signal strengths from TXNIP+ neutrophils, SPP1+ macrophages, M1- and M2-like macrophages, respectively. **J,** The contribution of each L-R pair to the overall CXCL signaling pathway among macrophage, neutrophil, and T-cell subtypes. **K–M,** Visualization of the cell-cell communication patterns mediated by the most significant L-R pairs in CXCL pathway. **N,** Heat map showing the relative strengths of the significant outgoing and incoming signaling patterns in all communication pathways with dominant sender and receiver immune cell subtypes. The top colored bar plot represents the sum of column of values displayed in the heat map representing the different cell populations. The right gray bar plots represents the sum of row of values, representing the different signaling pathways.

To elucidate how cells coordinate different pathways to drive communication, we investigated the global communication patterns between the different immune cell populations. We identified four outgoing patterns (Fig. 4A) and four incoming patterns (Fig. 4B). Outgoing signals from CD4^+^ T cells formed the largest communication pattern, with autocrine MHC-II and PECAM signaling observed (Fig. 4A and B). Outgoing signals from CD8^+^ T cells converge on the CLEC pathway, which is autocrine, together with signals from ANNEXIN, CD99, and IFN-II pathways. Tregs send signals along the LCK and VCAM pathways and are recipient to CD86 signaling. The IL1β signaling pathway is the most prominent outgoing pathway for neutrophils, which is also autocrine (Fig. 4A and B). Neutrophils are influenced by CXCL ligands and ICAM signals from CD4^+^ T cells, ANNEXIN signals from CD8^+^ T cells, and ADREG5 signals most likely from other cells in the metastatic microenvironment not explored here. CD4^+^ T cells receive signals from Tregs through the VCAM pathway, as well as signals along the CD45, CCL, and ITGB2 pathways. CD8^+^ T cells are influenced by LCK signals from Tregs, and additional signals along the CD99 and MHC-I signaling pathways. Finally, we compared the overall signaling roles of CD8^+^ and CD4^+^ T cells in CRCPT and LM. Our analysis revealed that in PT, CD8^+^ T cells are prominent senders, whereas the CD4^+^ T cells are prominent receivers. In CRCLM, these roles are strikingly reversed (Fig. 4C).

### Macrophages Communicate with T Cells and TXNIP+ Neutrophils Through MHC and CXCL Pathways in CRCLM

We then characterized the neutrophil subtypes in CRCLM to investigate their communication patterns with T cell and macrophage populations from the same dataset, focusing on the immunosuppressive TXNIP+ neutrophils and SPP1+ macrophages (18). We identified 11 additional neutrophil subtypes in CRCLM (Fig. 4D; Supplementary Tables S8 and S14): Inflammation regulatory (Inf_reg) neutrophils expressing genes important for inflammatory regulation (*TMG2*, *CCL4*, *CCL3*, and *PI3*), COX+ neutrophils expressing glycolysis genes (*DYNLL1*, *COX20*, and *ENO1*), IFN+ neutrophils enriched for IFN response genes (*ISG15*, *IFIT3*, *IFIT1*, and *IFITM3*), ARG1+ neutrophils expressing canonical neutrophil markers (*MMP9 and S100A12*) together with the T-cell suppressive markers *ARG1* and *TXNIP*, HLA-DR+ neutrophils expressing several genes from the HLA family (*Hla-drb1, hla-dra*), activated neutrophils enriched for markers of neutrophil activation (*DEFA3*, *CAMP*, *LTF*, *MMP8*) and HSP+, MT+ and RPS+ neutrophils highly expressing heat shock, mitochondrial and ribosomal proteins, respectively and PLPP3+ neutrophils expressing genes that converge on JAK-STAT and EGFR signaling (*PLPP3*, *FNIP2*, *PLIN2*, *SNAPC1*, *CSTB*, *CTSD*, *VEGFA*) and has been reported in other scRNA-seq studies of neutrophils (15, 37).

Analysis of the communication network between neutrophil subtypes, T cells and macrophages confirmed the recipient role of CD8^+^ T cells in the CRCLM microenvironment (Fig. 4E) with signals from TXNIP+ neutrophils specifically targeting CD8^+^ T cells (Fig. 4F). Macrophages exhibited diverse communication patterns, interacting with all other immune cell subtypes with stronger interactions with CD4^+^ and CD8^+^ T cells. This was observed for all macrophage subtypes, where subtypes were defined by the markers used in the original publications (Fig. 4G–I). The significant L-R interactions with highest communication probabilities from M1- and M2-like macrophages, proliferating MKI67+ and SPP1+ macrophages targeting CD4^+^ and CD8^+^ T cells were through the MHC-II and MHC-I pathways, respectively (Supplementary Fig. S6A–S6D). Both MKI67+ and SPP1+ macrophages exhibited stronger communication probabilities with M1-and M2-like macrophages along the MIF-(CD74/CXCR4) and MIF-(CD74/CXCR2) axes (Supplementary Fig. S5C and S5D). Both M1-like and SPP1+ macrophages showed the highest communication probability with TXNIP+ neutrophils through the CXCL8-CXCR2 L-R interaction (Supplementary Fig. S5A and S5D). Further analysis of the CXCL pathway identified CXCL8-CXCR2 as the major L-R interaction (Fig. 4J), with additional outgoing signals from other neutrophil subtypes targeting the TXNIP+ population (Fig. 4K). M2-like macrophages communicate with all immune cell populations investigated here through CXCL12–CXCR4 interactions (Fig. 4L) whereas signals from both M1-like and SPP1+ macrophages target TXNIP+ and IFN+ neutrophils through CXCL2–CXCR2 interactions (Fig. 4M). Finally, analysis of the aggregated cell-cell communication network from all signaling pathways identified both macrophages and CD4^+^ T cells as dominant senders in CRCLM (Fig. 4N). Collectively, our data confirm the importance of the CXCL8/CXCR2 axis in immune cell interactions and highlight the dominant roles of macrophages and CD4^+^ T cells within the immunosuppressive CRCLM environment.

###  Neutrophils Influence T-cell Suppression in CRCLM

To recapitulate the microenvironmental influences on neutrophils, we stimulated WT neutrophils with both GMCSF, which has been shown to be produced by mouse *Kras^G12D/+^* mutant tumors (38) and IL1β, the major influence on lineage generation we had observed. IL1β stimulation induced upregulation of *Txnip* expression (Supplementary Fig. S7A; Supplementary Table S2) but not *Cxcr2* expression (Supplementary Fig. S7B) suggesting a possible autocrine loop in this context. Neutrophils stimulated with GMCSF showed higher levels of both *Txnip* (Supplementary Fig. S7C) and *Cxcr2* expression suggesting an influence of tumor produced chemokines on neutrophil lineage development (Supplementary Fig. S7D).

**FIGURE 5:**
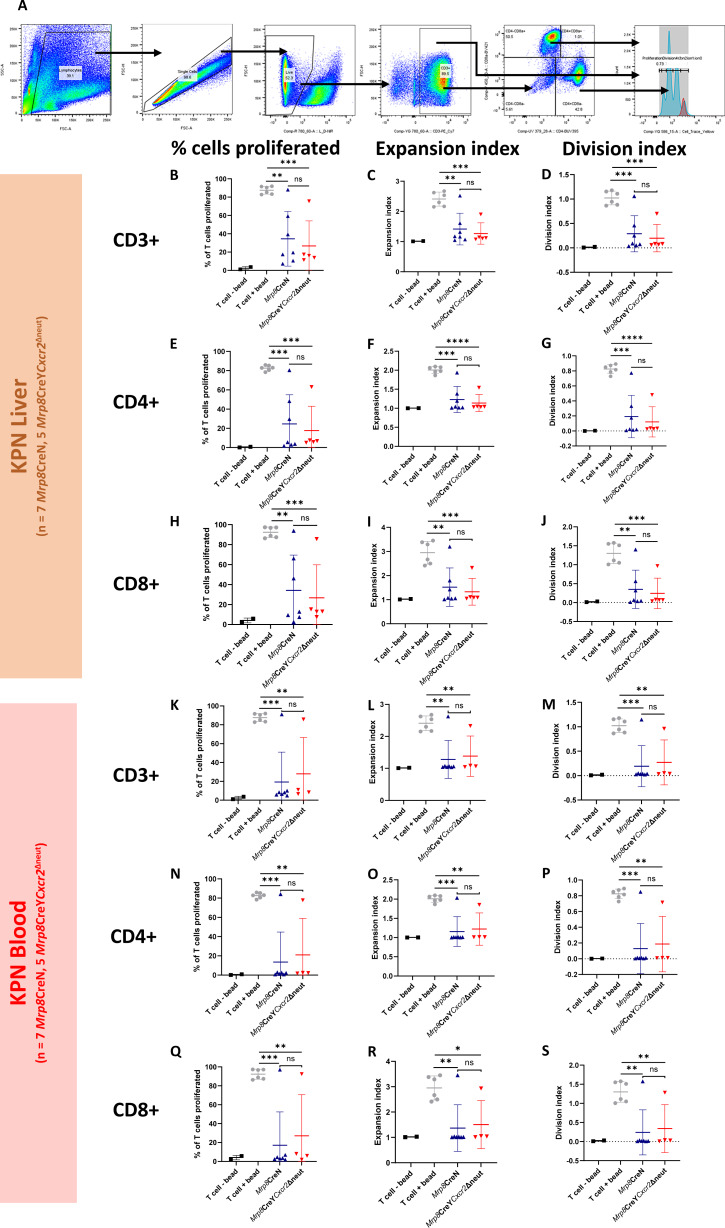
Loss of neutrophil-specific CXCR2 does not alter suppression of T-cell proliferation in neutrophils from the metastatic niche in mice bearing tumors. Neutrophils from the liver and blood of littermate mice either *Mrp8-Cre-Cxcr2^+/+^* (*Mrp8*CreN) or *Mrp8-Cre-Cxcr2*^fl/fl^ (*Mrp8*CreY*Cxcr2*Δneut) mice were harvested at clinical endpoint 32-33 days following intrasplenic injection of villinCreER *Kras*^G12D/+^*Trp53^f^*^l/fl^*Rosa26*^N1CD/+^ (KPN) cells derived from digestion of organoids from a single organoid line. These were placed in coculture with T cells from WT mice with CD3/CD28 stimulating beads and proliferation of CD3^+^ CD4^+^ and CD8^+^ T cells, and analyzed through flow cytometry 40 hours later. **A,** Flow cytometry gating of T cells and neutrophils from coculture. Cells were gated on FSC-A/SSC-A (not shown), FCS-A/FSC-H for singlets, Live/Dead, CD3, and CD4/CD8 to define CD3^+^, CD3^+^/CD4^+^, and CD3^+^/CD8^+^ populations for analysis. Cell trace yellow allows for tracking of T-cell proliferation. The signal for each T-cell division becomes subsequently dimmer, and allows for calculation of the number of T-cell generations. Gating obtained from stimulated T cell only control (blue) and unstimulated T cell control (red). **B,** Proportion of CD3^+^ T cells that proliferated in coculture with *Mrp8*CreN and *Mrp8*CreY*Cxcr2*Δneut neutrophils from tumor-bearing livers (**B**–**J**, cocultures from 7 *Mrp8*CreN mice and 5 *Mrp8*CreY*Cxcr2*Δneut mice). **C,** Expansion index of CD3^+^ T cells in coculture with *Mrp8*CreN and *Mrp8*CreY*Cxcr2*Δneut neutrophils from tumor-bearing livers. **D,** Division index of CD3^+^ T cells in coculture with *Mrp*8CreN and *Mrp8*CreY*Cxcr2*Δneut neutrophils from tumor-bearing livers. **E,** Proportion of CD4^+^ T cells that proliferated in coculture with *Mrp8*CreN and *Mrp8*CreY*Cxcr2*Δneut neutrophils from tumor-bearing livers. **F,** Expansion index of CD4^+^ T cells in coculture with *Mrp8*CreN and *Mrp8*CreY*Cxcr2*Δneut neutrophils from tumor-bearing livers. **G,** Division index of CD4^+^ T cells in coculture with *Mrp8*CreN and *Mrp8*CreY*Cxcr2*Δneut neutrophils from tumor-bearing livers. **H,** Proportion of CD8^+^ T cells that proliferated in coculture with *Mrp8*CreN and *Mrp8*CreY*Cxcr2*Δneut neutrophils from tumor-bearing livers. **I,** Expansion index of CD8^+^ T cells in coculture with *Mrp8*CreN and *Mrp8*CreY*Cxcr2*Δneut neutrophils from tumor-bearing livers. **J,** Division index of CD8^+^ T cells in coculture with *Mrp8*CreN and *Mrp8*CreY*Cxcr2*Δneut neutrophils from tumor-bearing livers. **K,** Proportion of CD3^+^ T cells that proliferated in coculture with *Mrp8*CreN and *Mrp8*CreY*Cxcr2*Δneut neutrophils from blood of tumor-bearing mice (**K**–**S,** 7 *Mrp8*CreN mice and 5 *Mrp8*CreY*Cxcr2*Δneut mice). **L,** Expansion index of CD3^+^ T cells that proliferated in coculture with *Mrp8*CreN and *Mrp8*CreY*Cxcr2*Δneut neutrophils from blood of tumor-bearing mice. **M,** Division index of CD3^+^ T cells that proliferated in coculture with *Mrp8*CreN and *Mrp8*CreY*Cxcr2*Δneut neutrophils from blood of tumor-bearing mice. **N,** Proportion of CD4^+^ T cells that proliferated in coculture with *Mrp8*CreN and *Mrp8*CreY*Cxcr2*Δneut neutrophils from blood of tumor-bearing mice. **O,** Expansion index of CD4^+^ T cells that proliferated in coculture with *Mrp8*CreN and *Mrp8*CreY*Cxcr2*Δneut neutrophils from blood of tumor-bearing mice. **P,** Division index of CD4^+^ T cells that proliferated in coculture with *Mrp8*CreN and *Mrp8*CreY*Cxcr2*Δneut neutrophils from blood of tumor-bearing mice. **Q,** Proportion of CD8^+^ T cells that proliferated in coculture with *Mrp8*CreN and *Mrp8*CreY*Cxcr2*Δneut neutrophils from blood of tumor-bearing mice. **R,** Expansion index of CD8^+^ T cells that proliferated in coculture with *Mrp8*CreN and *Mrp8*CreY*Cxcr2*Δneut neutrophils from blood of tumor-bearing mice. **S,** Division index of CD8^+^ T cells that proliferated in coculture with *Mrp8*CreN and *Mrp8*CreY*Cxcr2*Δneut neutrophils from blood of tumor-bearing mice. *, *P* < 0.05 on unpaired *t* test. **, *P <* 0.01 on unpaired *t* test.

Furthermore, tumors producing CXCR2 and CXCR1 chemokine receptor agonists induce neutrophil extracellular traps (NET), which pose a level of functional impairment on cytotoxic T or natural killer cells (39). To investigate the potential mechanisms through which our proposed T-cell suppressive neutrophil subtypes may impair T-cell function, we performed T-cell proliferation assays (gating strategy shown in Fig. 5A) and cocultured WT and CXCR2fl neutrophils with KPN organoids to assess the presence of extracellular DNA (which is released in NETosis), neutrophil activation, and neutrophil maturity.

Neutrophils sorted from the livers of villin-Cre^ERT2^*Kras^G12D/+^ Trp53*^fl/fl^*Rosa26*^N1icd/+^ (KPN) tumor-bearing mice 28 days following intrasplenic injection of Mrp8-Cre-Cxcr2^fl/fl^ (*Mrp8*CreYCxcr2Δneut) or Mrp8-Cre-negative-Cxcr2^fl/fl^ (*Mrp8*CreN) backgrounds showed significant suppression of T-cell proliferation in the Cxcr2^fl/fl^ setting comparable with WT neutrophils (Fig. 5B–D). This effect was exerted on both CD4^+^ (Fig. 5E–G) and CD8^+^ T cells (Fig. 5H–J). This effect was seen in CD3^+^, CD4^+^, or CD8^+^ T cells in coculture with neutrophils from blood from tumor-bearing mice (Fig. 5K–S). Coculture of KPN organoids and neutrophils (flow gating strategy in Supplementary Fig. S8A and S8B) revealed no influence on organoid viability (Supplementary Fig. S8C), neutrophil activation (Supplementary Fig. S8D) or presence of extracellular DNA seen in NETosis (Supplementary Fig. S8E) in this context when compared with *Mrp8*CreN neutrophils. This suggests that CXCR2 is not required to induce the maximum immunosuppressive effect seen in tumor-associated neutrophils within the CRCLM microenvironment and does not alter neutrophil phenotype in health.

## Discussion

There is a need to identify novel targets for therapy in metastatic colorectal cancer to circumvent resistance to current treatments (40). Previous work we have performed has suggested a clear role for neutrophils enhancing metastatic progression of KRAS-mutant cancer, across different tumor types (3, 41). Here, we have used publicly available scRNA-seq datasets and data generated in-house to explore single-cell transcriptomic profiles and assess differences in neutrophil transcriptomic states in health, PTs, and metastases across different diseases. By considering these cells in the context of the microenvironment, we gain insight into how the microenvironment is regulated.

We show that across species, mouse and human, and tissue types, healthy and tumor-specific neutrophil populations can be identified at single-cell transcriptomic level. We demonstrate that in health, neutrophils exhibit different transcriptomic signatures according to the tissue they are derived from. This is also true in cancer, where we demonstrate two distinct transcriptomic signatures observed in PT. In CRCLM, tumor-associated neutrophils exhibit the same signatures as the primary site; however, these populations are heterogeneous, encompassing additional, distinct transcriptomic changes. Our findings support the role of the tumor microenvironment in recruiting and transforming neutrophils into more immunosuppressive phenotypes (42). The major interactions between the different immune cell populations in CRCLM are summarized in Supplementary Fig. S9.

Recent studies have highlighted the roles of opposing neutrophil phenotypes, antitumorigenic N1 or protumorigenic N2, that exacerbate the progression of cancer depending on their prevalence (13), however, with advances in understanding of plasticity of neutrophils these states appear oversimplified, with neutrophils likely responsive to both the tissue of residence and microenvironmental signaling of the tumor and associated stromal cells. These populations were largely defined on the basis of their function and no cell surface markers have been identified thus far to differentiate between the two (43). In this study, we established the transcriptomic signatures of two distinct neutrophil subtypes in PTs: H_enriched and T_enriched neutrophil subtypes, which are conserved across species and across different cancers.

Neutrophils within CRCLM include a subset enriched for genes implicated in T-cell expansion, survival, and activation (30, 34). This metastatic-specific neutrophil signature identified in human CRCLM was equally present in a murine CRCLM bulk-RNA-seq dataset generated in our laboratory. Through the TNF pathway, tumor-associated neutrophils induce CD8^+^ T-cell apoptosis, further exacerbating their immunosuppressive phenotype (44). Moreover, S100A8-expressing neutrophils facilitate metastasis through the suppression of CD8^+^ T cells (45). Here, we show an upregulation of TNF signaling in metastatic neutrophils, concomitant with the overexpression of S100A8 and G0S2, implicated in the positive regulation of apoptosis. As such, we propose the presence of a metastasis-specific neutrophil subtype that specifically targets T cells to suppress them.

Our pseudotime analysis suggests a developmental trajectory of neutrophils that progresses from the healthy subtype to the tumor-specific population and finally a metastasis-specific population; a lineage that is largely driven by IL1β/CXCL8/CXCR2 axis. We show that tumor-associated neutrophils not only respond to IL1β/CXCR2 in their environment but equally signal through IL1β and CXCR2 in an autocrine fashion. When stimulated by IL1β we see upregulation of *Txnip* in neutrophils, but not *Cxcr2*. Interestingly, GMCSF, produced by KRAS mutant tumors (38), increases expression of both *Txnip* and *Cxcr2* compared with controls. The IL1’/CXCL8/CXCR2 axis has been implicated in several tumor types and plays a role in neutrophil recruitment (5, 46). The genetic ablation of *Cxcr2* in mice eliminates tumor accumulation and enhances T-cell infiltration and function (47). Moreover, targeting CXCR2+ immunosuppressive neutrophils, either independently or in combination with additional treatments, enhances antitumor immune activity; specifically, that of CD8^+^ T cells, and reduces tumor burden across different cancer types (48). Here, we provide data that supports the role of neutrophil-specific CXCR2 expression in enhancing colorectal liver metastasis in KRAS mutant cancer. Our data suggest education of these neutrophils by the microenvironment, in particular CD4^+^ T cells and a direct regulation of CD8^+^ T cells. T-cell proliferation assays in the tumor-bearing setting demonstrated that loss of neutrophil-specific *Cxcr2* does not attenuate the immunosuppressive function of neutrophils in the metastatic niche. Collectively, our analysis suggests that *Cxcr2* is important to neutrophil function in the tumor-bearing setting within CRCLM, and this stimulus may be derived from tumor cell and microenvironmentally produced chemokines. Stimulation experiments support transcriptomic analyses suggesting the importance of the IL1β/TXNIP/CXCR2 axis in supporting the development of metastatic specific neutrophils with potential to enhance T-cell suppression. This supports the presence of a CXCR2+ T-cell supressing neutrophil subset in CRCLM, the elimination of which can enhance T-cell infiltration and function.

It is important to account for tumor stage when considering the tumor-suppressive effect of targeting CXCR2. We propose that targeting IL1β independently or in combination with CXCR2, could be more favorable at earlier stages, where it may hinder the progression of neutrophils toward the tumor-specific subtype, permitting re-education of neutrophils to a tumor-killing phenotype, in addition to permitting an opportunity for other tumor-directed therapies. Late-stage interventions could target the CXCR2+ T-cell suppressive neutrophil subtypes through utilizing CXCR2 antagonists in combination with immune checkpoint inhibitors to counteract T-cell exhaustion. However, the dynamic nature of the tumor microenvironment should not be overlooked and the potential temporal shifts in the abundance and dominance of certain neutrophil subtypes should be considered during tumor progression. Our findings also implicate the TNF pathway in CRCLM associated neutrophil populations, as well as LTB, complement system and antigen presentation pathways in CD4^+^ T cells from the same tissue, highlighting the potential of harnessing these aspects of the tumor microenvironment to selectively activate neutrophils for immunotherapy (7).

Finally, we demonstrate that the transcriptomic signature of CD4^+^ T cells is altered in CRCLM. They are drivers of the signaling network in CRCLM and their interaction with neutrophils, CD8^+^ T cells and Tregs is essential to mediate immunosuppression as summarized in Fig. 4D. CD8^+^ and CD4^+^ T cells receive signals along the MHC-I and MHC-II pathways respectively, from macrophages and presumably due to direct interactions with tumor cells. The direct effects of IFN-II on T cells are largely suppressive (49), thus, we hypothesize that incoming IFN-II signals from CD8^+^ T cells may drive the suppression of CD4^+^ T cells in metastasis, specifically the cytotoxic subtype. Among the outgoing signaling pathways from CD4^+^ T cells was GALECTIN. The upregulation of Galectin-9 by IFN-II has an apoptosis-inducing activity in both CD4^+^ and CD8^+^ T cells; with CD8^+^ T cells being more susceptible (50). We identified two autocrine signaling pathways in CD4^+^ and CD8^+^ T cells: PECAM1 and CLEC, respectively. The adhesion molecule PECAM1 inhibits T-cell function in mice through the effects of TGFβ (51) and the C-type lectin receptor CLEC-1 negatively regulates antigen cross-presentation by dendritic cells to CD8^+^ T cells (52), supporting our hypothesis of diminished T-cell activity in the metastatic environment.

We demonstrate that neutrophils receive immunosuppressive signals from both CD4^+^ and CD8^+^ T cells. ANNEXIN signaling elicits proinvasive and protumoral properties in a number of cancers, whereby neutrophil microvesicles enriched in Annexin A1 and TGFβ are immunosuppressive (53). ICAM1 expression immobilizes neutrophils and enhances their migration and infiltration (54). Several L-R interactions in the CXCL pathway were between neutrophils, macrophages, and CD4^+^ T cells, suggesting an additional role of CD4^+^ T cells and macrophages in driving the immunosuppressive neutrophil phenotype. Autocrine IL1β-IL1βR2 and CXCL8–CXCL2 interactions within neutrophils support our pseudotime analysis. Neutrophils are recipients to ADGRE5 signaling, which has a role in tumor invasion and metastasis (55), potentially reflecting tumor–neutrophil interactions. We predict that Tregs receive CD86 signals, which upon their engagement with CTLA-4 receptor hamper the antigen-presenting ability of antigen-presenting cells to activate T cells (56). They primarily suppress CD4^+^ and CD8^+^ T cells via the VCAM and LCK pathways, respectively. VCAM1 is essential for T-cell extravasation and the Src-kinase LCK plays a critical role in initiating and regulating T-cell receptor signaling, whereby LCK inhibition selectively depletes effector Tregs and increases memory CD8^+^ T cells (57).

### Study Limitations

The integration of multiple scRNA-seq datasets allow for the systematic comparison of different cell types across different cancers; however, the approach still holds several shortcomings owing to inevitable batch effects due to different cell isolation methods, tissue handling protocols, library preparation, and sequencing platforms that remain sources of undesired biological and technical variability.

Our results implicate the IL1β/CXCL8/CXCR2 axis in the developmental trajectory of neutrophils; however, the validation of the interaction between these molecules and neutrophil behavior was beyond the primarily computational scope of this study. Additional upregulation of TXNIP in this context has not been previously described and requires further study as to its role and regulation of these processes. We have previously shown that treating KPN mice with a CXCR2 small-molecule inhibitor had no effect on either survival or PT burden but rather reduced metastasis profoundly. Moreover, targeted depletion of neutrophils resulted in an increase of infiltrating CD8^+^ T cells within the metastatic niche (5) which is in line with our findings. Future work should address the effects of targeting IL1β to investigate how this molecule alters neutrophil behavior and polarization.

In conclusion, there exist two neutrophil transcriptomic subtypes that predominate in PTs and are conserved across human and mouse cancers. We propose a developmental trajectory progressing from healthy neutrophils toward a tumor-specific subtype in PTs, with heterogeneous expression profiles of neutrophils present within metastases. However, a T-cell suppressive neutrophil lineage can be identified in CRCLM that specifically interacts with CD8^+^ T cells. This lineage is largely driven by the IL1β/CXCL8/CXCR2 axis. The metastatic niche further fosters an immunosuppressive environment, through the interplay between neutrophils, macrophages, CD8^+^ T cells, CD4^+^ T cells, and Tregs. CD4^+^ T cells and macrophages are the dominant signal senders and regulators of the immunosuppressive microenvironment with CD8-positive T cells largely receiving signals. As such, these interactions, and their timings should be considered when developing future immunotherapy trials in CRCLM.

## Supplementary Material

Supplementary Figure S1 Figure S1. Neutrophil proportions in KPN CRC models and in integrated mouse neutrophil dataset.

Supplementary Figure S2 Figure S2. Gene expression and neutrophil signatures in neutrophils from healthy and tumour tissue.

Supplementary Figure S3 Figure S3. Scoring for the M_enriched signature in NSCLC primary tumour dataset and outgoing signals from cell populations in CRCLM.

Supplementary Figure S4 Figure S4. cell-cell communication networks at a signalling pathway level.

Supplementary Figure S5 Figure S5. L-R interactions from the significant signalling pathways that mediate neutrophil and T-cell communication in CRCLM.

Supplementary Figure S6 Figure S6. L-R interactions from the significant signalling pathways that mediate communication between macrophages and other immune-cell subtypes in CRCLM.

Supplementary Figure S7 Figure S7. Exogenous application of IL-1β and GM-CSF alter transcription of Cxcr2 and Txnip in wild type neutrophils ex vivo.

Supplementary Figure S8 Figure S8. CXCR2-lacking neutrophils from healthy mice exhibit no functional differences from wild type in co-culture with KPN organoids

Supplementary Figure S9 Figure S9: Summary of findings.

Supplementary Tables Table S1: Mouse data for ScRNAsequencing
Table S2: Primer sequences for qPCR
Table S3. Top 5 markers in the integrated mouse dataset neutrophil clusters
Table S4: Pseudotime Lineages of the Neutrophil datasets
Table S5: Lineage-specific differentialy expressed genes (Start Vs End) in PYMT dataset.
Table S6: Lineage-specific differentialy expressed genes (Start Vs End) in CRC dataset.
Table S7: Lineage-specific differentialy expressed genes (Start Vs End) in human NSCLC dataset.
Table S8. Top 10 markers in the human CRCLM dataset neutrophil clusters
Table S9: Top 10 Differentially expressed genes in neutrophils derived from primary tumors and metastatic tissue.
Table S10: Differentially expressed genes in CRCLM neutrophils
Table S11: Differentially expressed genes in CD4 T cells in LM vs PT
Table S12: Differentially expressed genes in CD8 T cells in LM vs PT
Table S13: Differentially expressed genes in CD4 T cells in LM vs PT used in KEGG analysis(Threshold >3)
Table S14: Description of the different neutrophil subtypes identified in CRCLM

## References

[1] Ballesteros I , Rubio-Ponce A, Genua M, Lusito E, Kwok I, Fernández-Calvo G, . Co-option of neutrophil fates by tissue environments. Cell 2020;183: 1282-97. 33098771 10.1016/j.cell.2020.10.003

[2] McFarlane AJ , Fercoq F, Coffelt SB, Carlin LM. Neutrophil dynamics in the tumor microenvironment. J Clin Invest 2021;131: e143759. 33720040 10.1172/JCI143759 PMC7954585

[3] Steele CW , Karim SA, Leach JDG, Bailey P, Upstill-Goddard R, Rishi L, . CXCR2 inhibition profoundly suppresses metastases and augments immunotherapy in pancreatic ductal adenocarcinoma. Cancer Cell 2016;29: 832-45. 27265504 10.1016/j.ccell.2016.04.014 PMC4912354

[4] White M , Tsantoulis P, Lannagan T, Najumudeen A, Ridgeway RA, Campbell AD, . 26P NOTCH1 driven metastasis in BRAF mutated colorectal cancer. Ann Oncol 2022;33.

[5] Jackstadt R , van Hooff SR, Leach JD, Cortes-Lavaud X, Lohuis JO, Ridgway RA, . Epithelial NOTCH signaling rewires the tumor microenvironment of colorectal cancer to drive poor-prognosis subtypes and metastasis. Cancer Cell 2019;36: 319-36. 31526760 10.1016/j.ccell.2019.08.003 PMC6853173

[6] Gungabeesoon J , Gort-Freitas NA, Kiss M, Bolli E, Messemaker M, Siwicki M, . A neutrophil response linked to tumor control in immunotherapy. Cell 2023;186: 1448-64. 37001504 10.1016/j.cell.2023.02.032 PMC10132778

[7] Linde IL , Prestwood TR, Qiu J, Pilarowski G, Linde MH, Zhang X, . Neutrophil-activating therapy for the treatment of cancer. Cancer Cell 2023;41: 356–72 . 36706760 10.1016/j.ccell.2023.01.002 PMC9968410

[8] Zhu G , Pei L, Xia H, Tang Q, Bi F. Role of oncogenic KRAS in the prognosis, diagnosis and treatment of colorectal cancer. Mol Cancer 2021;20: 143. 34742312 10.1186/s12943-021-01441-4 PMC8571891

[9] Kargl J , Busch SE, Yang GHY, Kim KH, Hanke ML, Metz HE, . Neutrophils dominate the immune cell composition in non-small cell lung cancer. Nat Commun 2017;8: 14381. 28146145 10.1038/ncomms14381 PMC5296654

[10] Steele CW , Whittle T, Joshua Smith J. Review: KRAS mutations are influential in driving hepatic metastases and predicting outcome in colorectal cancer. Chin Clin Oncol 2019;8: 53. 31597434 10.21037/cco.2019.08.16

[11] Neutrophils are critical to the effectiveness of cancer immunotherapy. Cancer Discov 2023;13: 1285. 10.1158/2159-8290.CD-RW2023-055 37057909

[12] Leslie J , Mackey JBG, Jamieson T, Ramon-Gil E, Drake TM, Fercoq F, . CXCR2 inhibition enables NASH-HCC immunotherapy. Gut 2022;71: 2093-106. 35477863 10.1136/gutjnl-2021-326259 PMC9484388

[13] Mizuno R , Kawada K, Itatani Y, Ogawa R, Kiyasu Y, Sakai Y. The role of tumor-associated neutrophils in colorectal cancer. Int J Mol Sci 2019;20: 529. 30691207 10.3390/ijms20030529 PMC6386937

[14] Hedrick CC , Malanchi I. Neutrophils in cancer: heterogeneous and multifaceted. Nat Rev Immunol 2022;22: 173-87. 34230649 10.1038/s41577-021-00571-6

[15] Zilionis R , Engblom C, Pfirschke C, Savova V, Zemmour D, Saatcioglu HD, . Single-cell transcriptomics of human and mouse lung cancers reveals conserved myeloid populations across individuals and species. Immunity 2019;50: 1317-34. 30979687 10.1016/j.immuni.2019.03.009 PMC6620049

[16] Grieshaber-Bouyer R , Radtke FA, Cunin P, Stifano G, Levescot A, Vijaykumar B, . The neutrotime transcriptional signature defines a single continuum of neutrophils across biological compartments. Nat Commun 2021;12: 2856. 34001893 10.1038/s41467-021-22973-9 PMC8129206

[17] Alshetaiwi H , Pervolarakis N, McIntyre LL, Ma D, Nguyen Q, Rath JA, . Defining the emergence of myeloid-derived suppressor cells in breast cancer using single-cell transcriptomics. Sci Immunol 2020;5: eaay6017. 32086381 10.1126/sciimmunol.aay6017 PMC7219211

[18] Wu Y , Yang S, Ma J, Chen Z, Song G, Rao D, . Spatiotemporal immune landscape of colorectal cancer liver metastasis at single-cell level. Cancer Discov 2022;12: 134-53. 34417225 10.1158/2159-8290.CD-21-0316

[19] Zhang L , Li Z, Skrzypczynska KM, Fang Q, Zhang W, O'Brien SA, . Single-cell analyses inform mechanisms of myeloid-targeted therapies in colon cancer. Cell 2020;181: 442-59. 32302573 10.1016/j.cell.2020.03.048

[20] Azizi E , Carr AJ, Plitas G, Cornish AE, Konopacki C, Prabhakaran S, . Single-cell map of diverse immune phenotypes in the breast tumor microenvironment. Cell 2018;174: 1293-08. 29961579 10.1016/j.cell.2018.05.060 PMC6348010

[21] Kuhns DB , Priel DAL, Chu J, Zarember KA. Isolation and functional analysis of human neutrophils. Curr Protoc Immunol 2015;111: 7.23.1-7.23.16. 10.1002/0471142735.im0723s111 PMC4668800 26528633

[22] Prame Kumar K , Nicholls AJ, Wong CHY. Partners in crime: neutrophils and monocytes/macrophages in inflammation and disease. Cell Tissue Res 2018;371: 551-65. 29387942 10.1007/s00441-017-2753-2 PMC5820413

[23] Aloe C , Wang H, Vlahos R, Irving L, Steinfort D, Bozinovski S. Emerging and multifaceted role of neutrophils in lung cancer. Transl Lung Cancer Res 2021;10: 2806-18. 34295679 10.21037/tlcr-20-760 PMC8264329

[24] Roper J , Tammela T, Cetinbas NM, Akkad A, Roghanian A, Rickelt S, . *In vivo* genome editing and organoid transplantation models of colorectal cancer and metastasis. Nat Biotechnol 2017;35: 569-76. 28459449 10.1038/nbt.3836 PMC5462879

[25] Passegué E , Wagner EF, Weissman IL. Jun B deficiency leads to a myeloproliferative disorder arising from hematopoietic stem cells. Cell 2004;119: 431-43. 15507213 10.1016/j.cell.2004.10.010

[26] Steele CW , Karim SA, Foth M, Rishi L, Leach JDG, Porter RJ, . CXCR2 inhibition suppresses acute and chronic pancreatic inflammation. J Pathol 2015;237: 85-97. 25950520 10.1002/path.4555 PMC4833178

[27] Honey K . CCL3 and CCL4 actively recruit CD8+ T cells. Nat Rev Immunol 2006;6: 427.

[28] Takahashi K , Koga K, Linge HM, Zhang Y, Lin X, Metz CN, . Macrophage CD74 contributes to MIF-induced pulmonary inflammation. Respir Res 2009;10: 33. 19413900 10.1186/1465-9921-10-33 PMC2681459

[29] McLaren AS , Fetit R, Wood CS, Falconer J, Steele CW. Single cell sequencing of neutrophils demonstrates phenotypic heterogeneity and functional plasticity in health, disease, and cancer. Chin Clin Oncol 2023;12: 18. 37081709 10.21037/cco-22-121

[30] Muri J , Thut H, Kopf M. The thioredoxin-1 inhibitor Txnip restrains effector T-cell and germinal center B-cell expansion. Eur J Immunol 2021;51: 115-24. 32902872 10.1002/eji.202048851

[31] Jaffer T , Ma D. The emerging role of chemokine receptor CXCR2 in cancer progression. Transl Cancer Res 2016;5.

[32] Xiong X , Liao X, Qiu S, Xu H, Zhang S, Wang S, . CXCL8 in tumor biology and its implications for clinical translation. Front Mol Biosci 2022;9: 723846. 35372515 10.3389/fmolb.2022.723846 PMC8965068

[33] Froehlich J , Versapuech M, Megrelis L, Largeteau Q, Meunier S, Tanchot C, . FAM65B controls the proliferation of transformed and primary T cells. Oncotarget 2016;7: 63215-25. 27556504 10.18632/oncotarget.11438 PMC5325358

[34] Mao P , Hever MP, Niemaszyk LM, Haghkerdar JM, Yanco EG, Desai D, . Serine/threonine kinase 17A is a novel p53 target gene and modulator of cisplatin toxicity and reactive oxygen species in testicular cancer cells. J Biol Chem 2011;286: 19381-91. 21489989 10.1074/jbc.M111.218040 PMC3103316

[35] Wu L , Awaji M, Saxena S, Varney ML, Sharma B, Singh RK. IL-17–CXC chemokine receptor 2 axis facilitates breast cancer progression by up-regulating neutrophil recruitment. Am J Pathol 2020;190: 222-33. 31654638 10.1016/j.ajpath.2019.09.016 PMC6943375

[36] Haim-Vilmovsky L , Henriksson J, Walker JA, Miao Z, Natan E, Kar G, . Mapping Rora expression in resting and activated CD4+ T cells. PLoS One 2021;16: e0251233. 34003838 10.1371/journal.pone.0251233 PMC8130942

[37] Kleinstein SE , McCorrison J, Ahmed A, Hasturk H, Van Dyke TE, Freire M. Transcriptomics of type 2 diabetic and healthy human neutrophils. BMC Immunol 2021;22: 37. 34134627 10.1186/s12865-021-00428-6 PMC8207744

[38] Pylayeva-Gupta Y , Lee KE, Hajdu CH, Miller G, Bar-Sagi D. Oncogenic kras-induced GM-CSF production promotes the development of pancreatic neoplasia. Cancer Cell 2012;21: 836-47. 22698407 10.1016/j.ccr.2012.04.024 PMC3721510

[39] Teijeira Á , Garasa S, Gato M, Alfaro C, Migueliz I, Cirella A, . CXCR1 and CXCR2 chemokine receptor agonists produced by tumors induce neutrophil extracellular traps that interfere with immune cytotoxicity. Immunity 2020;52: 856-71. 32289253 10.1016/j.immuni.2020.03.001

[40] Shan J , Han D, Shen C, Lei Q, Zhang Y. Mechanism and strategies of immunotherapy resistance in colorectal cancer. Front Immunol 2022;13: 1016646. 36238278 10.3389/fimmu.2022.1016646 PMC9550896

[41] Jackstadt R , van Hooff SR, Leach JD, Cortes-Lavaud X, Lohuis JO, Ridgway RA, . Epithelial NOTCH signaling rewires the tumor microenvironment of colorectal cancer to drive poor-prognosis subtypes and metastasis. Cancer Cell 2019;36: 319-36. 31526760 10.1016/j.ccell.2019.08.003 PMC6853173

[42] Blanter M , Gouwy M, Struyf S. Studying neutrophil function *in vitro*: cell models and environmental factors. J Inflammation Res 2021;14: 141-62. 10.2147/JIR.S284941 PMC7829132 33505167

[43] Ohms M , Möller S, Laskay T. An Attempt to polarize human neutrophils toward N1 and N2 phenotypes *in**vitro*. Front Immunol 2020;11: 532. 32411122 10.3389/fimmu.2020.00532 PMC7198726

[44] Michaeli J , Shaul ME, Mishalian I, Hovav AH, Levy L, Zolotriov L, . Tumor-associated neutrophils induce apoptosis of non-activated CD8 T-cells in a TNFα and NO-dependent mechanism, promoting a tumor-supportive environment. Oncoimmunology 2017;6: e1356965. 29147615 10.1080/2162402X.2017.1356965 PMC5674962

[45] Wagner NB , Weide B, Gries M, Reith M, Tarnanidis K, Schuermans V, . Tumor microenvironment-derived S100A8/A9 is a novel prognostic biomarker for advanced melanoma patients and during immunotherapy with anti-PD-1 antibodies. J Immunother Cancer 2019;7: 343. 31806053 10.1186/s40425-019-0828-1 PMC6896585

[46] Raza S , Rajak S, Tewari A, Gupta P, Chattopadhyay N, Sinha RA, . Multifaceted role of chemokines in solid tumors: from biology to therapy. Semin Cancer Biol 2022;86: 1105-21. 10.1016/j.semcancer.2021.12.011 PMC7613720 34979274

[47] Steele CW , Karim SA, Leach JDG, Bailey P, Upstill-Goddard R, Rishi L, . CXCR2 inhibition profoundly suppresses metastases and augments immunotherapy in pancreatic ductal adenocarcinoma. Cancer Cell 2016;29: 832-45. 27265504 10.1016/j.ccell.2016.04.014 PMC4912354

[48] Gulhati P , Schalck A, Jiang S, Shang X, Wu CJ, Hou P, . Targeting T cell checkpoints 41BB and LAG3 and myeloid cell CXCR1/CXCR2 results in antitumor immunity and durable response in pancreatic cancer. Nat Cancer 2023;4: 62-80. 36585453 10.1038/s43018-022-00500-z PMC9925045

[49] Whitmire JK , Tan JT, Whitton JL. Interferon-γ acts directly on CD8+ T cells to increase their abundance during virus infection. J Exp Med 2005;201: 1053-9. 15809350 10.1084/jem.20041463 PMC2213135

[50] Yang R , Sun L, Li CF, Wang YH, Yao J, Li H, . Galectin-9 interacts with PD-1 and TIM-3 to regulate T cell death and is a target for cancer immunotherapy. Nat Commun 2021;12: 832. 33547304 10.1038/s41467-021-21099-2 PMC7864927

[51] Newman DK , Fu G, Adams T, Cui W, Arumugam V, Bluemn T, . The adhesion molecule PECAM-1 enhances the TGF-β-mediated inhibition of T cell function. Sci Signal 2016;9: ra27. 26956486 10.1126/scisignal.aad1242 PMC5087802

[52] Drouin M , Saenz J, Gauttier V, Evrard B, Teppaz G, Pengam S, . CLEC-1 is a death sensor that limits antigen cross-presentation by dendritic cells and represents a target for cancer immunotherapy. Sci Adv 2022;8: eabo7621. 36399563 10.1126/sciadv.abo7621 PMC9674301

[53] Araújo TG , Mota STS, Ferreira HSV, Ribeiro MA, Goulart LR, Vecchi L. Annexin A1 as a regulator of immune response in cancer. Cells 2021;10: 2245. 34571894 10.3390/cells10092245 PMC8464935

[54] Yang L , Froio RM, Sciuto TE, Dvorak AM, Alon R, Luscinskas FW. ICAM-1 regulates neutrophil adhesion and transcellular migration of TNF-α-activated vascular endothelium under flow. Blood 2005;106: 584-92. 15811956 10.1182/blood-2004-12-4942 PMC1635241

[55] Aust G , Zheng L, Quaas M. To detach, migrate, adhere, and metastasize: CD97/ADGRE5 in cancer. Cells 2022;11: 1538. 35563846 10.3390/cells11091538 PMC9101421

[56] Dees S , Ganesan R, Singh S, Grewal IS. Regulatory T cell targeting in cancer: emerging strategies in immunotherapy. Eur J Immunol 2021;51: 280-91. 33302322 10.1002/eji.202048992

[57] Li C , Jiang P, Wei S, Xu X, Wang J. Regulatory T cells in tumor microenvironment: new mechanisms, potential therapeutic strategies and future prospects. Mol Cancer 2020;19: 116. 32680511 10.1186/s12943-020-01234-1 PMC7367382

