# Correction: Characterizing Neutrophil Subtypes in Cancer Using scRNA Sequencing Demonstrates the Importance of IL1β/CXCR2 Axis in Generation of Metastasis-Specific Neutrophils

**DOI:** 10.1158/2767-9764.CRC-25-0159

**Published:** 2025-04-11

**Authors:** Rana Fetit, Alistair S. McLaren, Mark White, Megan L. Mills, John Falconer, Xabier Cortes-Lavaud, Kathryn Gilroy, Tamsin R.M. Lannagan, Rachel A. Ridgway, Colin Nixon, Varushka Naiker, Renee Njunge, Cassie J. Clarke, Declan Whyte, Kristina Kirschner, Rene Jackstadt, Jim Norman, Leo M. Carlin, Andrew D. Campbell, Owen J. Sansom, Colin W. Steele

In the original version of this article (1), the way the data in Fig. 5 were presented was incorrect. The data were presented as 4v6 replicates but should have been presented as 2v3 biological replicates. All experiments were repeated with the same organoid cell line, increasing the number of mice to ensure adequate power. In doing so, the authors found that neutrophils are immunosuppressive in this context. However, CXCR2 expression did not influence suppression of T cell proliferation as originally reported. Also, the gating of neutrophils in Supplementary Fig. S8 was inaccurate due to the axes being misinterpreted at the cell sorting stage. The experiments were repeated, as shown in Supplementary Fig. S8, using correctly sorted CD48^−^/Ly6G+ cells.

Figure 5 has been corrected using the new data, and errors in the Abstract, Introduction, Results, and Discussion related to the interpretation of the result of the T cell suppression due to neutrophil presence have been removed or modified in the latest online HTML and pdf versions of the article. Supplementary Fig. S8 has been replaced with a corrected version in the latest online HTML version of the article. The authors regret these errors.

## Supplementary Material

Supplementary Figure S8 Correction to Supplementary Figure 8
