# Supplementary material for: Characterizing Neutrophil Subtypes in Cancer Using scRNA Sequencing Demonstrates the Importance of IL1β/CXCR2 Axis in Generation of Metastasis-specific Neutrophils
Source: Cancer Res Commun. 2024 Feb 29;4(2):588–606. doi: 10.1158/2767-9764.CRC-23-0319 (PMC10903300; doi:10.1158/2767-9764.CRC-23-0319)
Supplement: Supplementary Figure S1 — Figure S1. Neutrophil proportions in KPN CRC models and in integrated mouse neutrophil dataset. [file crc-23-0319-s01.pdf]

SUPPLEMENTARY FIGURES

Figure S1

A

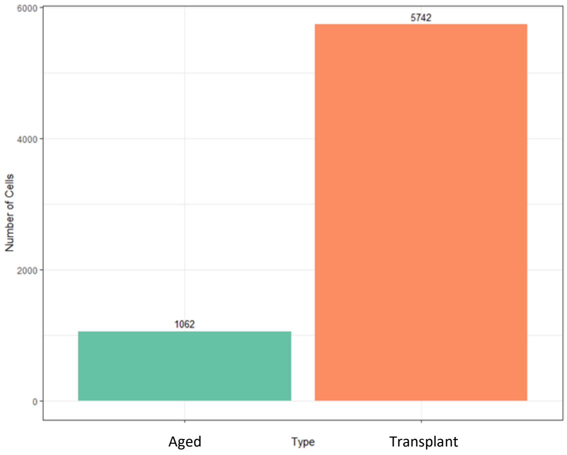

B

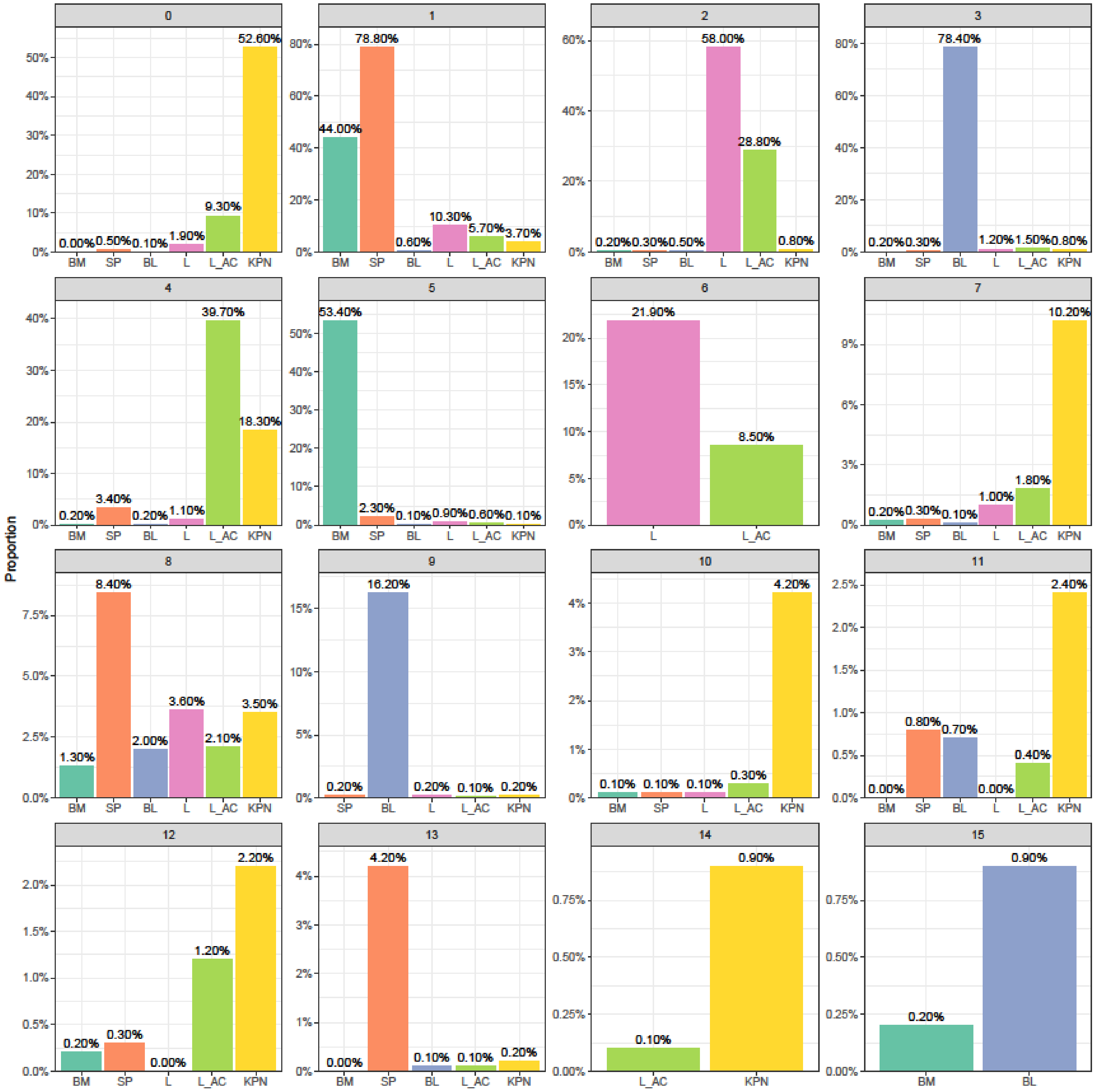

Figure S1. Neutrophil proportions in KPN CRC models and in integrated mouse neutrophil dataset.

(A) Proportion of neutrophils derived from aged GE mice and transplant models of CRC.

(B) Proportion of clusters (from figure 1B) in every tissue type in integrated mouse neutrophil dataset. BM: healthy bone marrow, SP: healthy spleen, BL: healthy blood, L: healthy lung, L_AC: lung adenocarcinoma, KPN: colorectal cancer with *Kras,Trp53* and Notch mutations.
