# Supplementary material for: Characterizing Neutrophil Subtypes in Cancer Using scRNA Sequencing Demonstrates the Importance of IL1β/CXCR2 Axis in Generation of Metastasis-specific Neutrophils
Source: Cancer Res Commun. 2024 Feb 29;4(2):588–606. doi: 10.1158/2767-9764.CRC-23-0319 (PMC10903300; doi:10.1158/2767-9764.CRC-23-0319)
Supplement: Supplementary Figure S2 — Figure S2. Gene expression and neutrophil signatures in neutrophils from healthy and tumour tissue. [file crc-23-0319-s02.pdf]

Figure S2

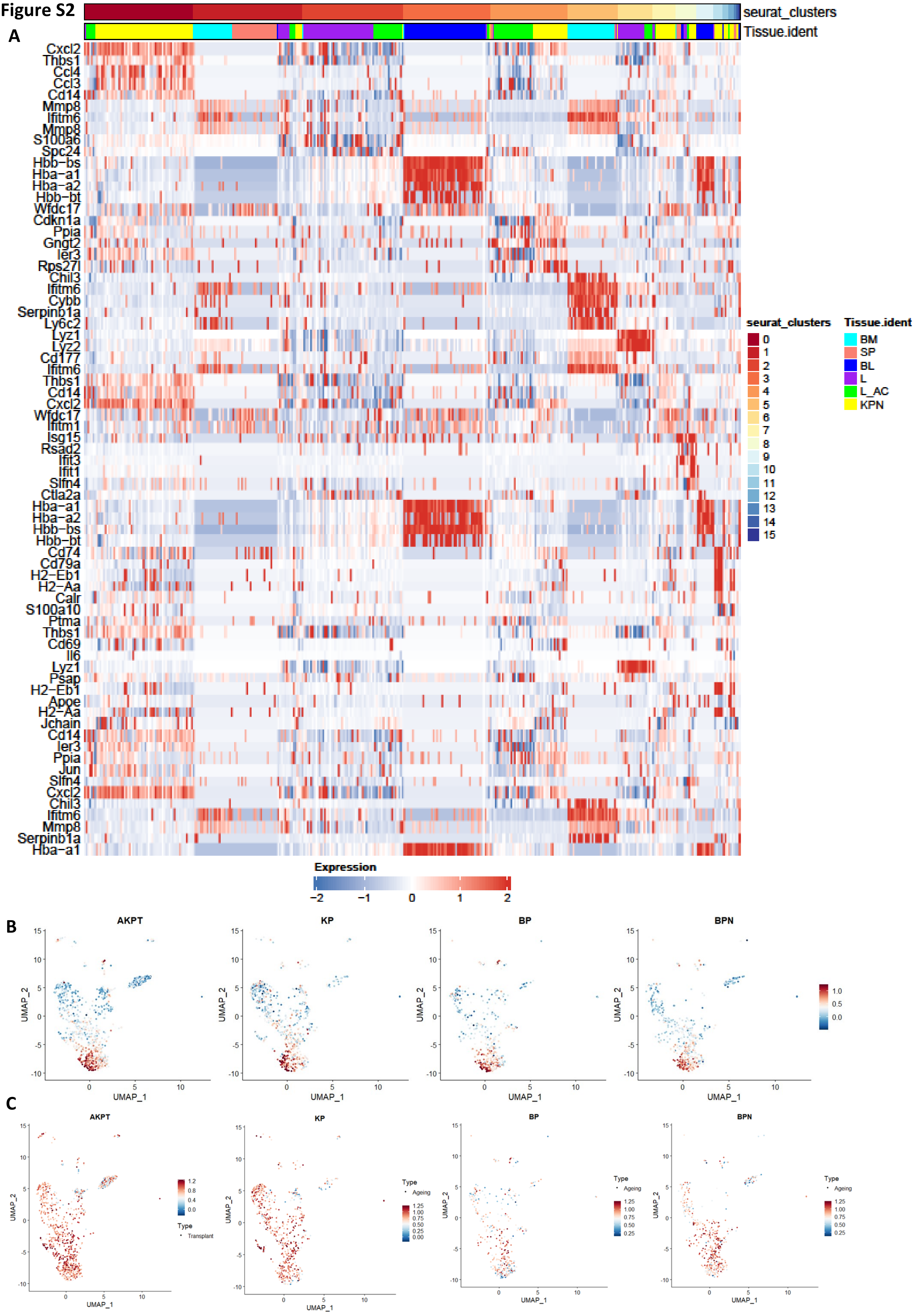
A
seurat_clusters
Tissue.ident
Cxcl2
Thbs1
Ccl4
Ccl3
Cd14
Mmp8
Ifitm6
Mmp8
S100a6
Spc24
Hbb−bs
Hba−a1
Hba−a2
Hbb−bt
Wfdc17
Cdkn1a
Ppia
Gngt2
Ier3
Rps27l
Chil3
Ifitm6
Cybb
Serpinb1a
Ly6c2
Lyz1
Lyz2
Cd177
Ifitm6
Thbs1
Cd14
Cxcl2
Wfdc17
Ifitm1
Isg15
Rsad2
Ifit3
Ifit1
Slfn4
Ctla2a
Hba−a1
Hba−a2
Hbb−bs
Hbb−bt
Cd74
Cd79a
H2−Eb1
H2−Aa
Calr
S100a10
Ptma
Thbs1
Cd69
Il6
Lyz1
Psap
H2−Eb1
Apoe
H2−Aa
Jchain
Cd14
Ier3
Ppia
Jun
Slfn4
Cxcl2
Chil3
Ifitm6
Mmp8
Serpinb1a
Hba−a1
seurat_clusters
0
1
2
3
4
5
6
7
8
9
10
11
12
13
14
15
Tissue.ident
BM
SP
BL
L
L_AC
KPN
Expression
−2
−1
0
1
2
B
AKPT
KP
BP
BPN
UMAP_2
UMAP_1
1.0
0.5
0.0
C
AKPT
KP
BP
BPN
UMAP_2
UMAP_1
1.2
0.8
0.4
0.0
Type
Transplant
Type
Ageing
1.25
1.00
0.75
0.50
0.25
0.00
Type
Ageing
1.25
1.00
0.75
0.50
0.25
Type
Ageing
1.25
1.00
0.75
0.50
0.25

Figure S2. Gene expression and neutrophil signatures in neutrophils from healthy and tumour tissue.

(A) Heatmap showing the top 5 markers of neutrophil clusters in the integrated mouse dataset. Top bar is coloured according to Seurat cluster, bottom bar is coloured according to tissue identity.

(B,C) Healthy and Tumour-specific neutrophil signatures are equally present in both GEM and transplant models of CRC listed in Table2.
