# Supplementary material for: Characterizing Neutrophil Subtypes in Cancer Using scRNA Sequencing Demonstrates the Importance of IL1β/CXCR2 Axis in Generation of Metastasis-specific Neutrophils
Source: Cancer Res Commun. 2024 Feb 29;4(2):588–606. doi: 10.1158/2767-9764.CRC-23-0319 (PMC10903300; doi:10.1158/2767-9764.CRC-23-0319)
Supplement: Supplementary Figure S3 — Figure S3. Scoring for the M_enriched signature in NSCLC primary tumour dataset and outgoing signals from cell populations in CRCLM. [file crc-23-0319-s03.pdf]

Figure S3

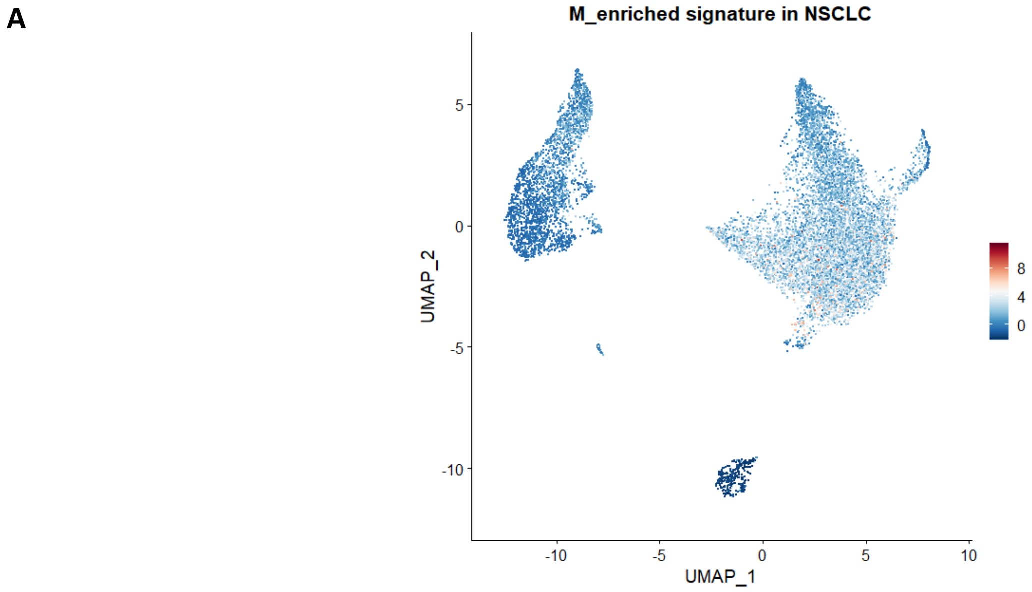

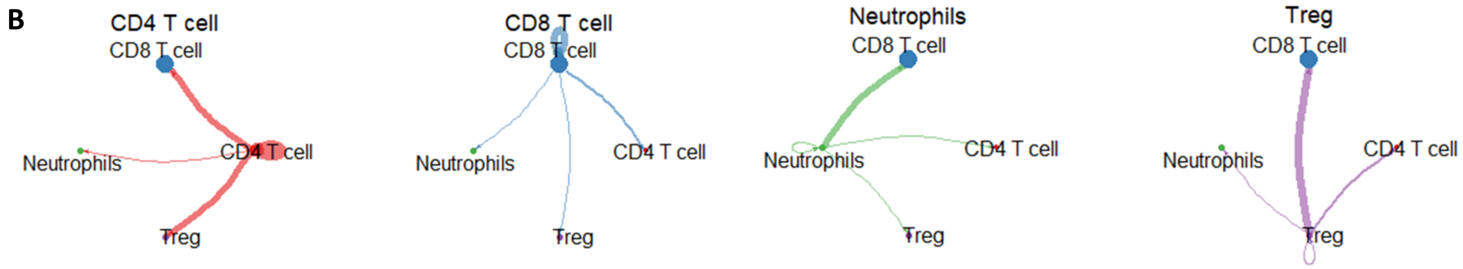

Figure S3. Scoring for the M_enriched signature in NSCLC primary tumour dataset and outgoing signals from cell populations in CRCLM.

(A) Gene scoring reveals that this signature is not enriched in the primary tumours, suggesting its specificity to the metastatic tumour dataset investigated in this study.

(B) Outgoing signals sent from each cell group in CRCLM.
