# Supplementary material for: Characterizing Neutrophil Subtypes in Cancer Using scRNA Sequencing Demonstrates the Importance of IL1β/CXCR2 Axis in Generation of Metastasis-specific Neutrophils
Source: Cancer Res Commun. 2024 Feb 29;4(2):588–606. doi: 10.1158/2767-9764.CRC-23-0319 (PMC10903300; doi:10.1158/2767-9764.CRC-23-0319)
Supplement: Supplementary Figure S4 — Figure S4. cell-cell communication networks at a signalling pathway level. [file crc-23-0319-s04.pdf]

Figure S4

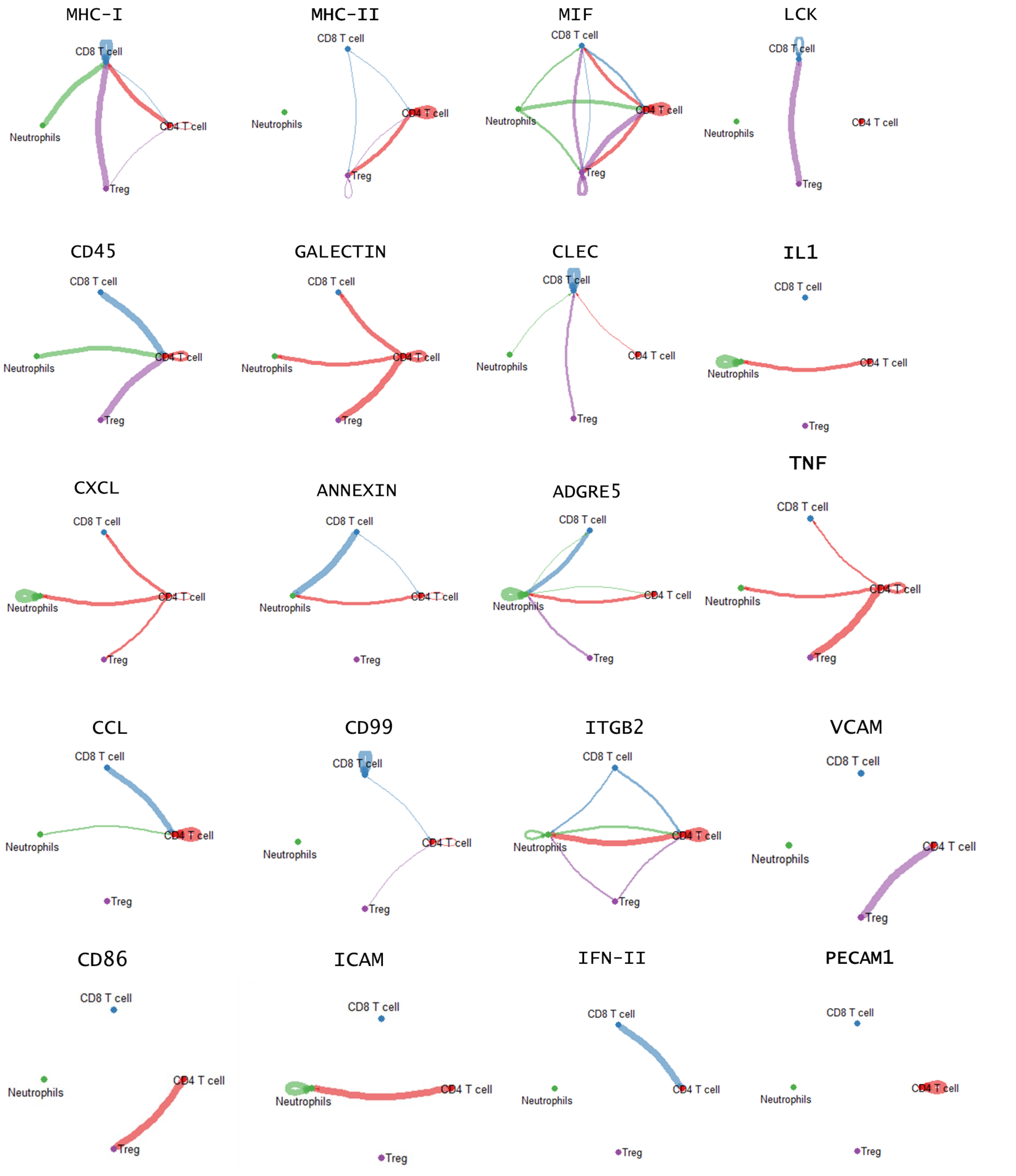

Figure S4. cell-cell communication networks at a signalling pathway level.

Chord plots showing the interaction strengths between the different immune populations in CRCLM across the 20 signalling pathways with significant communications.
