# Supplementary material for: Characterizing Neutrophil Subtypes in Cancer Using scRNA Sequencing Demonstrates the Importance of IL1β/CXCR2 Axis in Generation of Metastasis-specific Neutrophils
Source: Cancer Res Commun. 2024 Feb 29;4(2):588–606. doi: 10.1158/2767-9764.CRC-23-0319 (PMC10903300; doi:10.1158/2767-9764.CRC-23-0319)
Supplement: Supplementary Figure S5 — Figure S5. L-R interactions from the significant signalling pathways that mediate neutrophil and T-cell communication in CRCLM. [file crc-23-0319-s05.pdf]

Figure S5

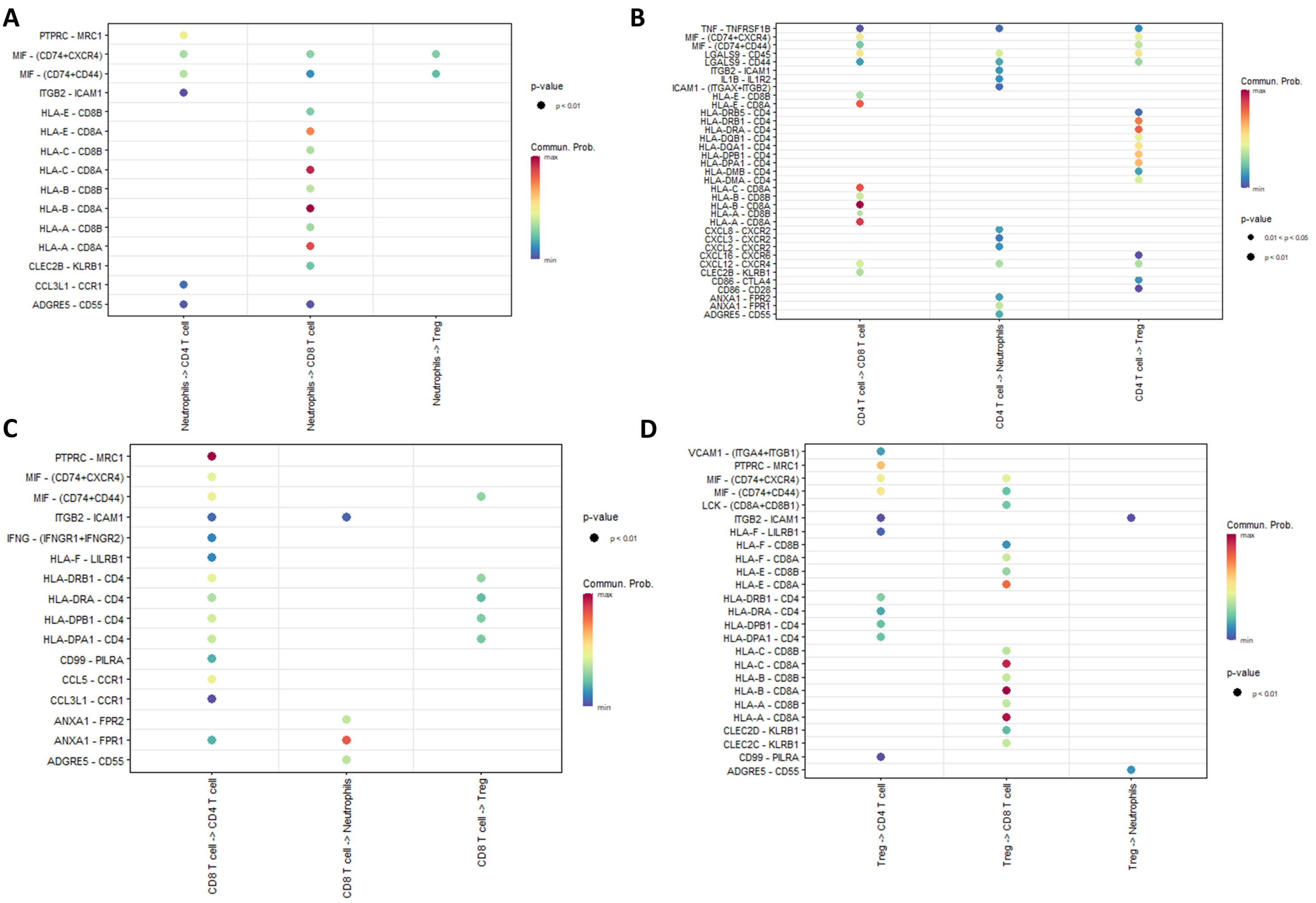

Figure S5. L-R interactions from the significant signalling pathways that mediate neutrophil and T-cell communication in CRCLM.

(A-D) Bubble plots showing significant L-R interactions and their communication probability across the 20 significant pathways from neutrophils, CD4+T-cells, CD8+T-cells and Treg cells to other cell groups.
