# Supplementary material for: Characterizing Neutrophil Subtypes in Cancer Using scRNA Sequencing Demonstrates the Importance of IL1β/CXCR2 Axis in Generation of Metastasis-specific Neutrophils
Source: Cancer Res Commun. 2024 Feb 29;4(2):588–606. doi: 10.1158/2767-9764.CRC-23-0319 (PMC10903300; doi:10.1158/2767-9764.CRC-23-0319)
Supplement: Supplementary Figure S6 — Figure S6. L-R interactions from the significant signalling pathways that mediate communication between macrophages and other immune-cell subtypes in CRCLM. [file crc-23-0319-s06.pdf]

Figure S6

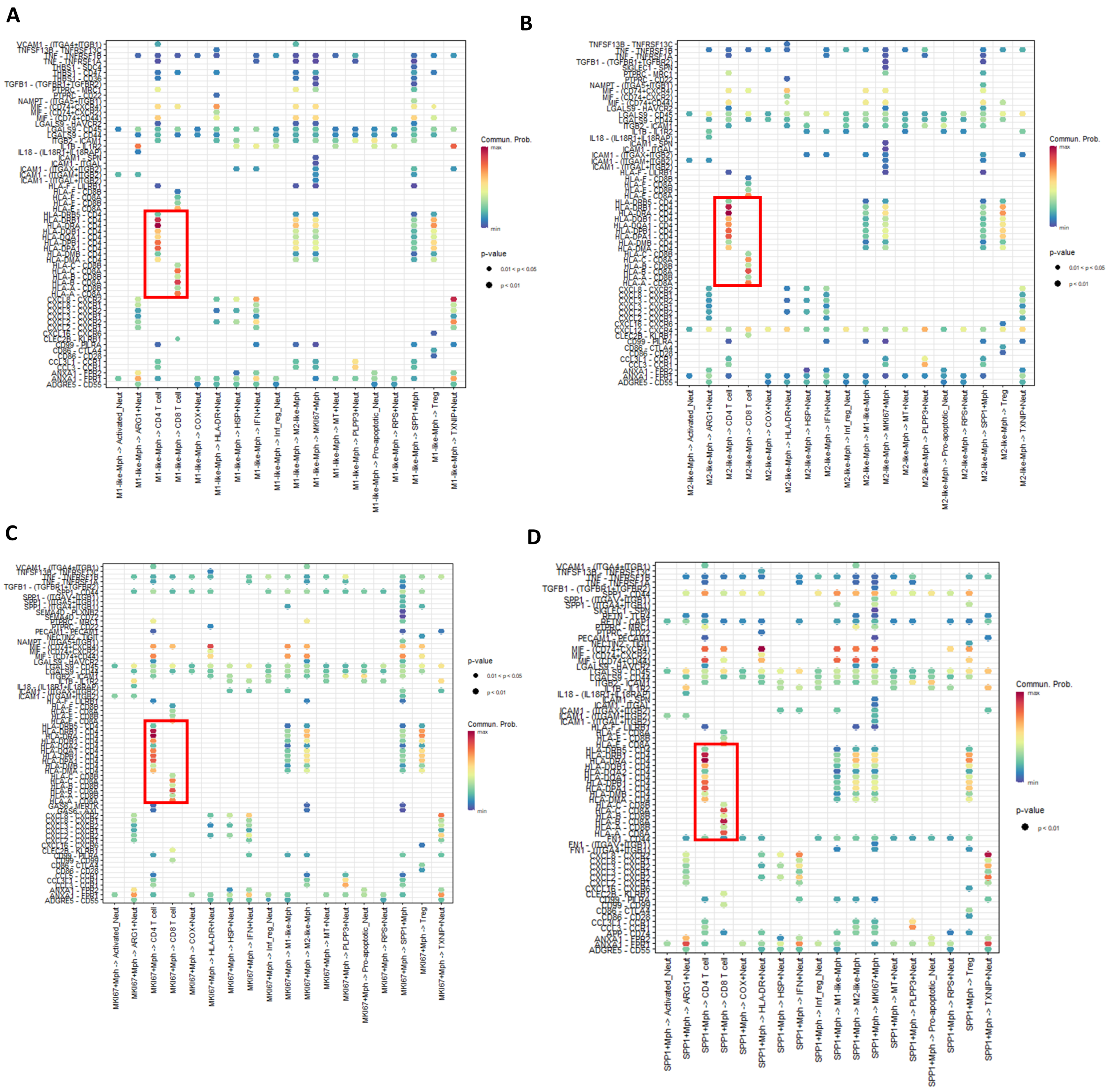

Figure S6. L-R interactions from the significant signalling pathways that mediate communication between macrophages and other immune-cell subtypes in CRCLM.

(A-D) Bubble plots showing significant L-R interactions and their communication probability across the significant pathways from M1-like, M2-like, MKI67+ and SPP1+ macrophages towards other immune-cell subtypes. Red boxes highlight the significant L-R interactions with highest communication probabilities targeting CD4+ and CD8+T-cells through MHC-II and MHC-I pathways.
