# Supplementary material for: Characterizing Neutrophil Subtypes in Cancer Using scRNA Sequencing Demonstrates the Importance of IL1β/CXCR2 Axis in Generation of Metastasis-specific Neutrophils
Source: Cancer Res Commun. 2024 Feb 29;4(2):588–606. doi: 10.1158/2767-9764.CRC-23-0319 (PMC10903300; doi:10.1158/2767-9764.CRC-23-0319)
Supplement: Supplementary Figure S7 — Figure S7. Exogenous application of IL-1β and GM-CSF alter transcription of Cxcr2 and Txnip in wild type neutrophils ex vivo. [file crc-23-0319-s07.pdf]

Figure S7

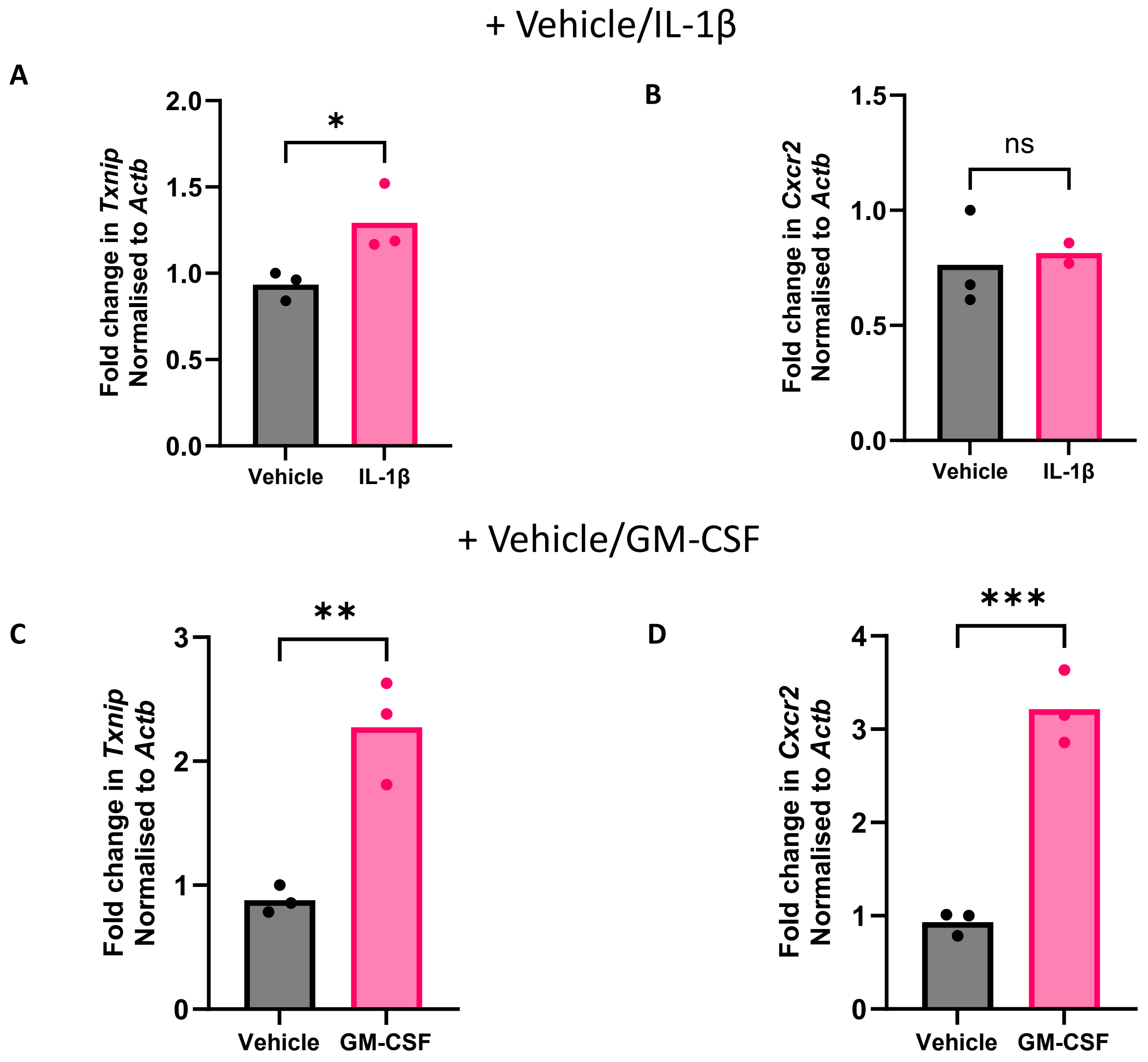

Figure S7. Exogenous application of IL-1β and GM-CSF alter transcription of *Cxcr2* and *Txnip* in wild type neutrophils ex vivo.

(A) Changes in *Txnip* expression measured by qPCR normalised to *Actb* following application of exogenous IL-1β to wild type (WT) mouse neutrophils (neutrophils from 3 mice used in both arms) .

(B) Changes in *Cxcr2* expression measured by qPCR normalised to *Actb* following application of exogenous IL-1β to wild type (WT) mouse neutrophils (n=3 mice, neutrophils used for both arms).

(C) Changes in *Txnip* expression measured by qPCR normalised to *Actb* following application of exogenous GM-CSF to wild type (WT) mouse neutrophils (n=3 mice, neutrophils used for both arms).

(D) Changes in *Cxcr2* expression measured by qPCR normalised to *Actb* following application of exogenous IL-1β to wild type (WT) mouse neutrophils (n=3 mice, neutrophils used for both arms).

* $p \leq 0.05$ on unpaired t test.

** $p \leq 0.01$ on unpaired t test.

***$p \leq 0.001$ on unpaired t test.

ns $p > 0.05$ on unpaired t test.
