# Supplementary material for: Characterizing Neutrophil Subtypes in Cancer Using scRNA Sequencing Demonstrates the Importance of IL1β/CXCR2 Axis in Generation of Metastasis-specific Neutrophils
Source: Cancer Res Commun. 2024 Feb 29;4(2):588–606. doi: 10.1158/2767-9764.CRC-23-0319 (PMC10903300; doi:10.1158/2767-9764.CRC-23-0319)
Supplement: Supplementary Figure S8 — Figure S8. CXCR2-lacking neutrophils from healthy mice exhibit no functional differences from wild type in co-culture with KPN organoids [file crc-23-0319-s08.pdf]

Figure S8

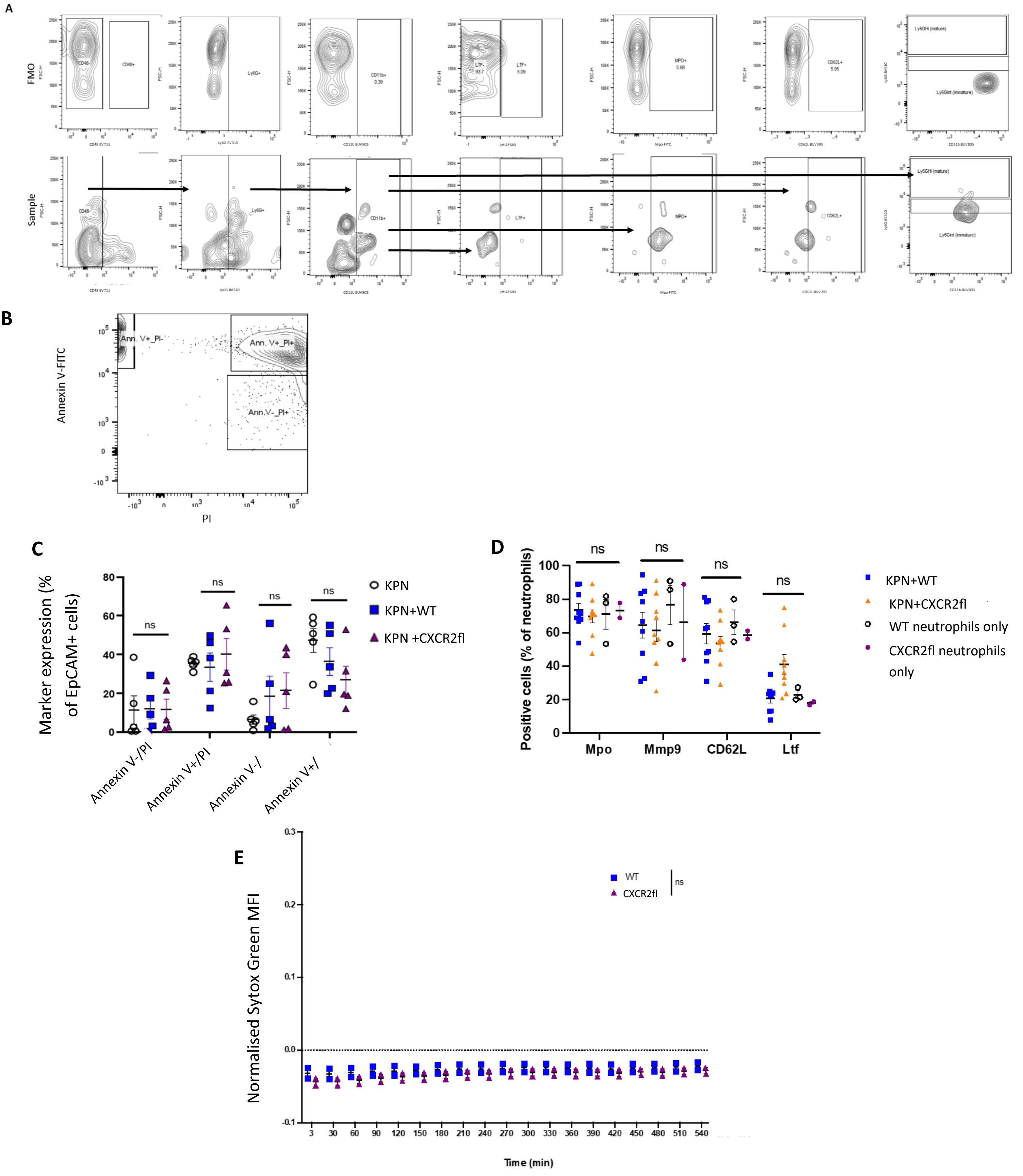

A
FMO
Sample
B
Annexin V-FITC
PI
Ann. V+_PI-
Ann. V+_PI+
Ann.V-_PI+
C
Marker expression (% of EpCAM+ cells)
ns
KPN
KPN+WT
KPN +CXCR2fl
Annexin V-/PI
Annexin V+/PI
Annexin V-/
Annexin V+/
D
Positive cells (% of neutrophils)
ns
Mpo
Mmp9
CD62L
Ltf
KPN+WT
KPN+CXCR2fl
WT neutrophils only
CXCR2fl neutrophils only
E
Normalised Sytox Green MFI
WT
CXCR2fl
ns
Time (min)

Figure S8. CXCR2-lacking neutrophils from healthy mice exhibit no functional differences from wild type in co-culture with KPN organoids

Mrp8-Cre-CXCR2$^{+/+}$ (WT) and *Mrp8-Cre-Cxcr2*$^{fl/fl}$ (CXCR2fl) neutrophils from healthy mice were assessed for functional changes using flow cytometry and time lapse microscopy during and following 11 hour co-culture with *villin*CreER *Kras*$^{G12D/+}$ *Trp*53$^{fl/fl}$ *Rosa26*$^{N1icd/+}$ (KPN) organoids.

(A) Gating strategy to assess neutrophil function following FCS-A/SSC-A for cells, FSC-A/FSC-H for singlets, and of CD45+ cells. Sample data from WT mouse.

(B) Gating strategy to assess organoid viability through levels of proprium iodide (PI) and annexin V following FCS-A/SSC-A for cells, FSC-A/FSC-H for singlets, selection of live cells following Live/Dead staining and of EpCAM+ cells. Annexin V positivity is associated with early apoptosis, and PI positivity with late apoptosis or necrosis. Viable cells are negative for both. Sample data from WT mouse.

(C) Assay of EpCAM+ cell flow cytometric measurement of annexin V and PI levels (n = 5 KPN only controls, 5 WT and 5 CXCR2fl from 4 WT and 4 CXCR2fl mice).

(D) Assay of flow cytometric measurement of neutrophil activation markers (n=8 KPN+WT, 9 KPN+CXCR2fl, 3 WT only controls, 2 CXCR2fl only controls from 4 WT and 4 CXCR2fl mice).

(E) Assay of presence of extracellular DNA in media, measured as mean fluorescence intensity (MFI) of Sytox Green normalised to control (n = 2 WT, 2 CXCR2fl from 2 WT and 2CXCR2fl mice, 3 technical replicates per condition and results pooled).

ns $p>0.05$ by two way ANOVA
