# Supplementary material for: Characterizing Neutrophil Subtypes in Cancer Using scRNA Sequencing Demonstrates the Importance of IL1β/CXCR2 Axis in Generation of Metastasis-specific Neutrophils
Source: Cancer Res Commun. 2024 Feb 29;4(2):588–606. doi: 10.1158/2767-9764.CRC-23-0319 (PMC10903300; doi:10.1158/2767-9764.CRC-23-0319)
Supplement: Supplementary Figure S9 — Figure S9: Summary of findings. [file crc-23-0319-s09.pdf]

Figure S9

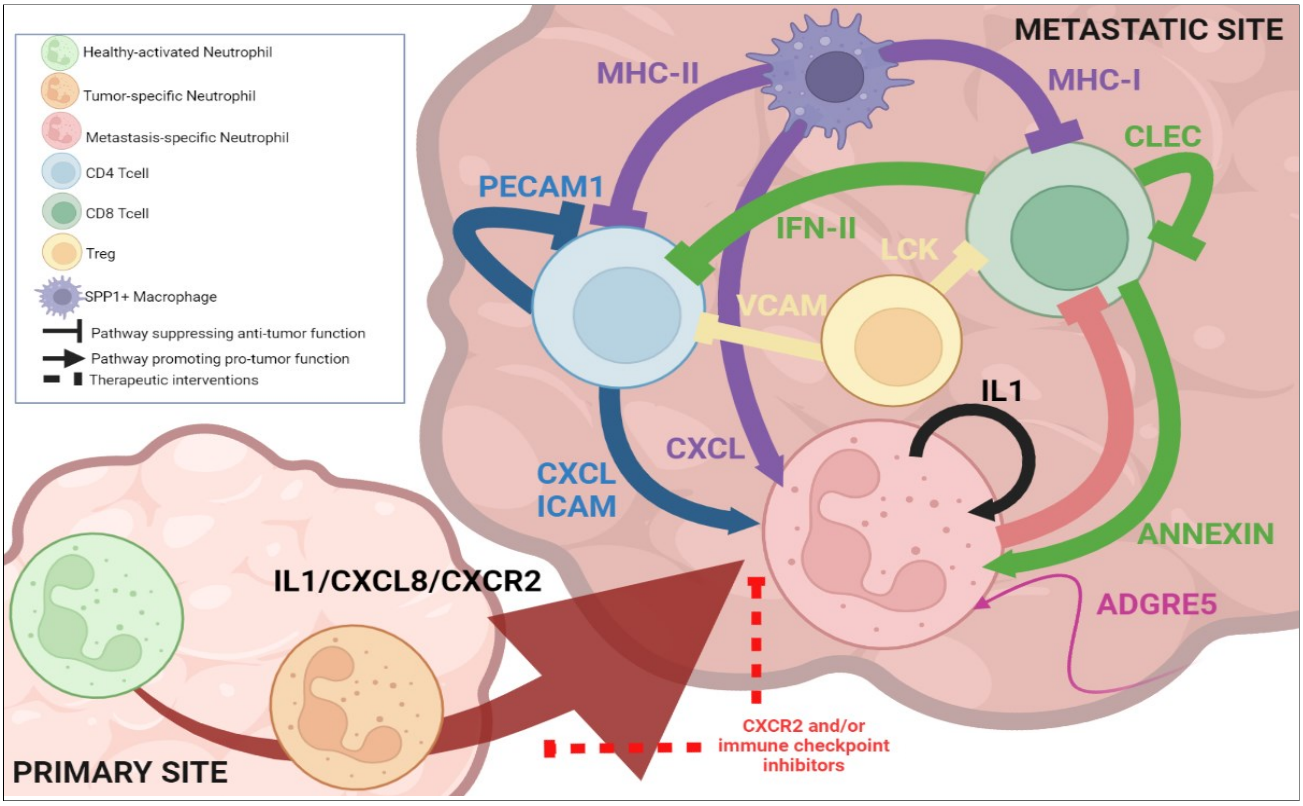

Summary of findings. IL1/CXCL8/CXCR2 axis drives neutrophil progression from health to cancer and malignancy. Several signalling pathways between neutrophils and other immune cells come together to foster an immunosuppressive, metastatic niche. Targeting different chemokines in along the IL1/CXCL8/CXCR2 will allow future stratification of treatments for targeting different neutrophil subtypes at early and late stages of cancer.
Figure created in biorender.com.
