# Supplementary material for: Correction: Characterizing Neutrophil Subtypes in Cancer Using scRNA Sequencing Demonstrates the Importance of IL1β/CXCR2 Axis in Generation of Metastasis-Specific Neutrophils
Source: Cancer Res Commun. 2025 Apr 11;5(4):609. doi: 10.1158/2767-9764.CRC-25-0159 (PMC11990819; doi:10.1158/2767-9764.CRC-25-0159)
Supplement: Supplementary Figure S8 — Correction to Supplementary Figure 8 [file crc-25-0159_supplementary_figure_s8_suppsf8.pptx]

## Slide 1
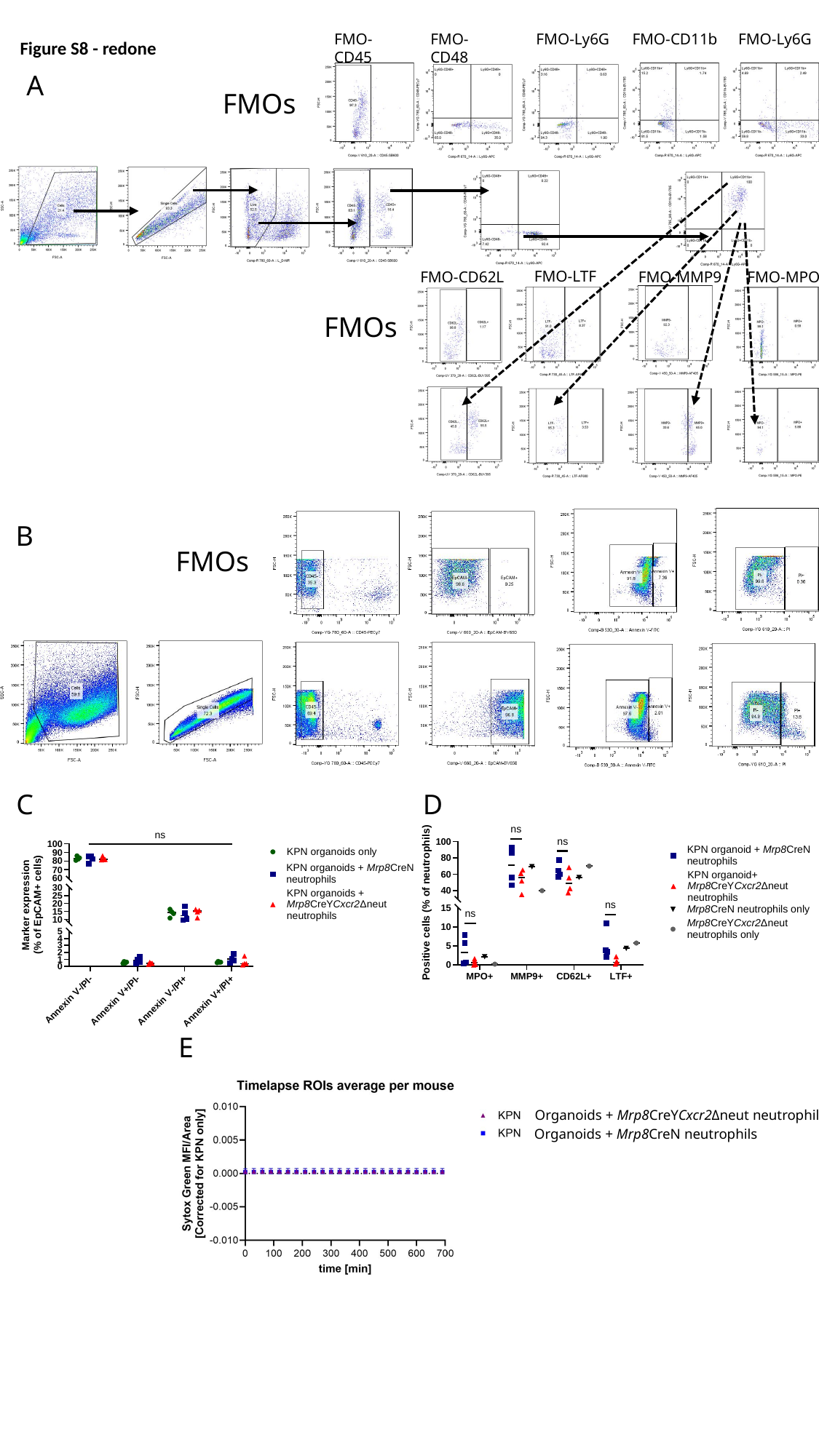

FMO-CD45
FMO-CD48
FMO-Ly6G
FMO-CD11b
FMO-Ly6G
Figure S8 - redone
A
FMOs
FMO-LTF
FMO-MMP9
FMO-CD62L
FMO-MPO
FMOs
B
FMOs
D
C
E
Organoids + Mrp8CreYCxcr2Δneut neutrophils
Organoids + Mrp8CreN neutrophils

## Slide 2
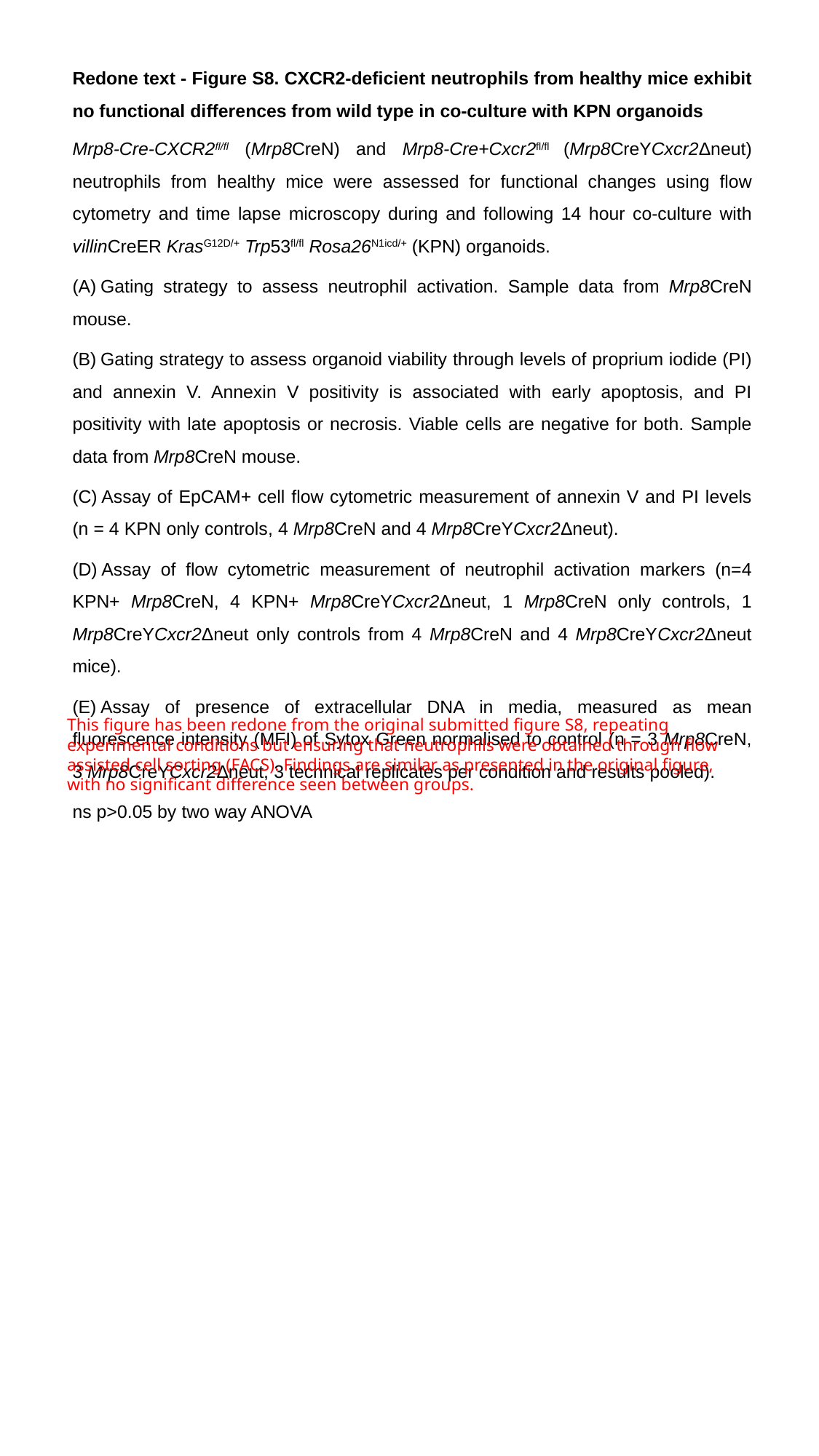

Redone text - Figure S8. CXCR2-deficient neutrophils from healthy mice exhibit no functional differences from wild type in co-culture with KPN organoids
Mrp8-Cre-CXCR2fl/fl (Mrp8CreN) and Mrp8-Cre+Cxcr2fl/fl (Mrp8CreYCxcr2Δneut) neutrophils from healthy mice were assessed for functional changes using flow cytometry and time lapse microscopy during and following 14 hour co-culture with villinCreER KrasG12D/+ Trp53fl/fl Rosa26N1icd/+ (KPN) organoids.
(A) Gating strategy to assess neutrophil activation. Sample data from Mrp8CreN mouse.
(B) Gating strategy to assess organoid viability through levels of proprium iodide (PI) and annexin V. Annexin V positivity is associated with early apoptosis, and PI positivity with late apoptosis or necrosis. Viable cells are negative for both. Sample data from Mrp8CreN mouse.
(C) Assay of EpCAM+ cell flow cytometric measurement of annexin V and PI levels (n = 4 KPN only controls, 4 Mrp8CreN and 4 Mrp8CreYCxcr2Δneut).
(D) Assay of flow cytometric measurement of neutrophil activation markers (n=4 KPN+ Mrp8CreN, 4 KPN+ Mrp8CreYCxcr2Δneut, 1 Mrp8CreN only controls, 1 Mrp8CreYCxcr2Δneut only controls from 4 Mrp8CreN and 4 Mrp8CreYCxcr2Δneut mice).
(E) Assay of presence of extracellular DNA in media, measured as mean fluorescence intensity (MFI) of Sytox Green normalised to control (n = 3 Mrp8CreN, 3 Mrp8CreYCxcr2Δneut, 3 technical replicates per condition and results pooled).
ns p>0.05 by two way ANOVA
This figure has been redone from the original submitted figure S8, repeating experimental conditions but ensuring that neutrophils were obtained through flow assisted cell sorting (FACS). Findings are similar as presented in the original figure, with no significant difference seen between groups.
